# A generic residue-numbering system for microbial rhodopsins

**DOI:** 10.1038/s44511-026-00004-w

**Published:** 2026-11-05

**Authors:** Flurin S. Hidber, Seiya Tajima, Koichiro E. Kishi, Yosuke Nishimura, Susumu Yoshizawa, Xavier Deupi, Hideaki E. Kato

**Affiliations:** 1 Condensed Matter Theory Group, PSI Center for Scientific Computing, Theory and Data, Villigen-PSI, Switzerland; 2 Department of Biology, ETH Zürich, Zürich, Switzerland; 3 Swiss Institute of Bioinformatics (SIB), Lausanne, Switzerland; 4 Research Center for Advanced Science and Technology, The University of Tokyo, Meguro, Tokyo Japan; 5 Department of Life Sciences, School of Arts and Sciences, The University of Tokyo, Meguro, Tokyo Japan; 6 Atmosphere and Ocean Research Institute, The University of Tokyo, Chiba, Japan; 7 Institute for Extra-cutting-edge Science and Technology Avant-garde Research of Life (X-star), Japan Agency for Marine-Earth Science and Technology (JAMSTEC), Kanagawa, Japan; 8 Graduate School of Frontier Sciences, The University of Tokyo, Chiba, Japan; 9 Collaborative Research Institute for Innovative Microbiology, The University of Tokyo, Tokyo, Japan; 10 Laboratory of Biomolecular Research, PSI Center for Life Sciences, Villigen-PSI, Switzerland; 11 Department of Biological Sciences, Graduate School of Science, The University of Tokyo, Bunkyo, Tokyo Japan; 12 FOREST, Japan Science and Technology Agency, Kawaguchi, Saitama Japan; 13 CREST, Japan Science and Technology Agency, Kawaguchi, Saitama Japan

**Keywords:** Biochemistry, Biophysics, Computational biology and bioinformatics, Microbiology, Neuroscience

## Abstract

Microbial rhodopsins are photoreceptors with a conserved seven-transmembrane core architecture that use the chromophore retinal—a vitamin A derivative—to convert light absorption into ion transport, signaling, and enzymatic activity. Since their discovery half a century ago, genome sequencing has uncovered thousands of these proteins across archaea, bacteria, eukaryotes, and viruses, revealing a diverse functional repertoire that includes ion pumps and channels, transducer activators, and enzyme/channel regulators. Although the structure of the seven-transmembrane bundle core and the retinal-binding pocket is highly conserved, the sequence identity across major classes is low. This low sequence identity, combined with the rapidly expanding number of microbial rhodopsins identified by metagenomics, makes direct comparisons across the superfamily increasingly difficult. Here, we introduce a generic residue-numbering system for microbial rhodopsins (MO numbering system), a structure-guided framework anchored to the retinal-binding pocket that enables consistent residue comparison across even the most divergent family members. Analyzing 129 structures and approximately 40,000 sequences, we demonstrate its utility by analyzing sequence–structure–function relationships across the superfamily, including previously uncharacterized members. The MO numbering system provides a common language for organizing the growing repertoire of microbial rhodopsins, integrating structural, spectroscopic, and electrophysiological data, and engineering next-generation optogenetic tools.

## Introduction

The microbial rhodopsin superfamily comprises light-sensitive proteins that use the chromophore retinal—a vitamin A derivative—to convert photon absorption into concerted cellular events. Since the discovery of the proton pump bacteriorhodopsin in *Halobacterium salinarum* (*Hs*BR) over fifty years ago^1^, microbial rhodopsins have revealed a striking diversity of functions. These proteins are found across all domains of life—Archaea, Bacteria, and Eukaryota— and viruses^2–4^, functioning as proton, chloride, or sodium pumps, cation and anion channels, activators of downstream signal transducers, and even as enzyme/channel/transporter regulators. Their photochemical properties are equally varied: while in most microbial rhodopsins light isomerizes all-*trans* retinal to the 13-*cis* form, some produce 7-*cis*, 9-*cis*, or 11-*cis* isomers as photoproducts^2,5–7^.

Despite this functional and photochemical diversity, all microbial rhodopsins share a common architecture: a tight bundle of seven transmembrane helices (TMs) surrounding the retinal-binding pocket, with retinal bound via a Schiff base to a conserved lysine on TM7. Some families contain an additional TM preceding TM1, whereas others exhibit an inverted membrane topology^6,8–10^. In addition, individual families adopt characteristic oligomeric assemblies, including dimers, trimers, pentamers, and hexamers^6,8,11–16^. Remarkably, this structural conservation persists even though sequence identity between major families can be as low as 10–12%^17^, with a nearly identical orientation of retinal in the binding pocket. Such conservation despite extreme sequence divergence^18^ suggests powerful evolutionary constraints on the architecture required for retinal binding and photoisomerization.

Microbial rhodopsins are often classified, annotated, and compared by tracking characteristic sequence motifs that are coupled to their function. However, the high sequence variability within the family complicates the analysis of large datasets obtained in metagenomics studies, particularly the comparison of distant subfamilies. Researchers in the field often resort to developing *ad hoc* sequence numbering systems using a reference rhodopsin, such as *Hs*BR, channelrhodopsin-2 from *Chlamydomonas reinhardtii* (*Cr*ChR2, a widely used optogenetics tool)^19^, or C1C2 also from *Chlamydomonas reinhardtii* (the first channelrhodopsin structure, a chimera between channelrhodopsin-1 and 2)^13^. This makes it challenging to recognize equivalent positions and conserved motifs in different rhodopsins throughout the scientific literature. Other fields have overcome this hurdle by adopting generic residue-numbering (GRN) systems. A prime example is the field of G protein-coupled receptors (GPCRs), in which the Ballesteros–Weinstein GRN system^20^—or its descendant GPCRdb system^21^—assigns a unique index to equivalent positions in different proteins. Similar systems developed for microbial rhodopsins are limited to a few specific families^22^ and cannot be applied across the entire superfamily.

In this work, we develop a GRN system for microbial opsins, termed the MO numbering system. This system is anchored primarily in the architecture of the retinal-binding pocket and the spatial proximity of residues to retinal, and is subsequently refined by incorporating sequence conservation, rather than relying solely on sequence conservation. This numbering system provides a consistent framework for comparing residues across even the most divergent rhodopsins. Using a dataset of 129 structures and approximately 40,000 sequences, we demonstrate its utility by mapping functionally important residues across ion pumps, channels, and sensory rhodopsins, and by identifying conserved sequence signatures in both characterized and previously uncharacterized rhodopsin families.

## Results

### Assembly of a dataset of microbial rhodopsin structures

**Fig. 1:**
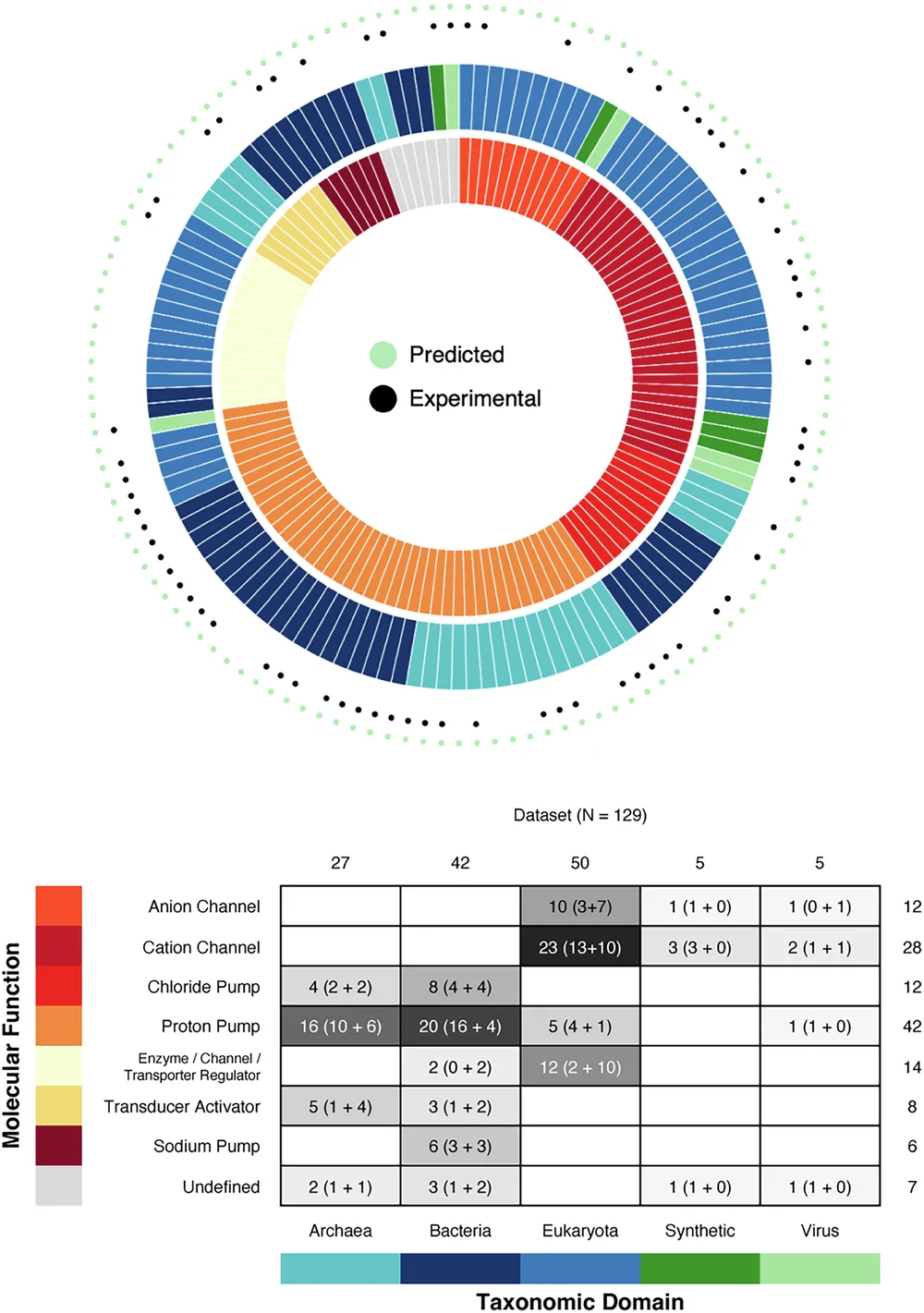
Dataset of microbial rhodopsin structures. The colors in the inner and outer rings indicate molecular function and taxonomic domain, respectively (as shown in the table). Experimental structures and computational models are marked with black and cyan circles, respectively. The table shows the composition of the dataset; each cell shows “total number of structures (experimental + predictions)” in that category.

Comparative structural analysis of microbial rhodopsins has been somewhat hindered by limited structural coverage of certain functional classes and taxonomic groups. To address this limitation, we assembled a comprehensive dataset of 129 structures, combining 69 experimentally determined structures and 60 computationally predicted models (Fig. 1). The dataset includes rhodopsins from all domains of life—archaea (27, ~21%), bacteria (42, ~33%), and eukaryota (50, ~39%)—and also includes synthetic variants (5, ~4%) and viral rhodopsins (5, ~4%). Functionally, the dataset spans proton pumps (42, ~33%), cation channels (28, ~22%), regulatory rhodopsins with fused domains (14, ~11%), anion channels (12, ~9%), chloride pumps (12, ~9%), transducer activators (8, ~6%), sodium pumps (6, ~5%), and rhodopsins of undefined function (7, 5%). Due to biases in available structural databases, proton pumps are overrepresented in the experimentally determined structures (31/69, ~45%). To balance functional coverage, we enriched underrepresented families through predictive structural modeling: enzyme/channel/transporter regulators (12 predictions), cation channels (11), proton pumps (11), anion channels (8), chloride pumps (6), transducer activators (6), sodium pumps (3), and rhodopsins of undefined function (3).

The dataset reflects known taxonomic distributions of rhodopsin functions. Channelrhodopsins—both cation-conducting (23/28) and anion-conducting (10/12)—are concentrated in eukaryotes, consistent with their restriction to algae and related protists^23^. Similarly, enzyme/channel/transporter regulatory rhodopsins are predominantly eukaryotic (12/14), consistent with enzyme rhodopsins having been identified mostly in fungi, green algae, and choanoflagellates^8^. Sodium pumps appear exclusively in bacteria (6/6), consistent with their known distribution in marine flavobacteria and related lineages^24^. Chloride pumps are found in both archaea and bacteria, reflecting independent evolutionary origins in these lineages^25^. Proton pumps show the broadest distribution across Archaea, Bacteria, and Eukaryota, consistent with ancestral sequence reconstruction studies suggesting that the last common ancestor of microbial rhodopsins functioned as a light-driven proton pump^26^.

### Validation of the structural predictions

As nearly half of our dataset consists of predicted models, we first assessed their accuracy using two complementary validation sets of experimental structures: a benchmark set (*n* = 41), composed of structures deposited before September 2021 (which may have been used in the training of the structure prediction methods), and a blind set (*n* = 27), composed exclusively of structures deposited after the model training cutoff. We assessed the accuracy of the models by comparing the protein backbone, the retinal-binding pocket, and retinal (Supplementary Fig. 1). The predicted models have good accuracy. Models in the benchmark set had a mean backbone Cα RMSD of 0.61 ± 0.23 Å, binding-pocket iRMSD of 0.51 ± 0.28 Å, and retinal L-RMSD of 0.40 ± 0.24 Å. Models in the blind set had a mean backbone Cα RMSD of 0.80 ± 0.24 Å, binding-pocket iRMSD of 0.75 ± 0.28 Å, and retinal L-RMSD of 0.62 ± 0.27 Å. The errors in the blind set are consistently higher, but the mean differences between the predicted models and the reference structures remain below 1 Å.

**Fig. 2:**
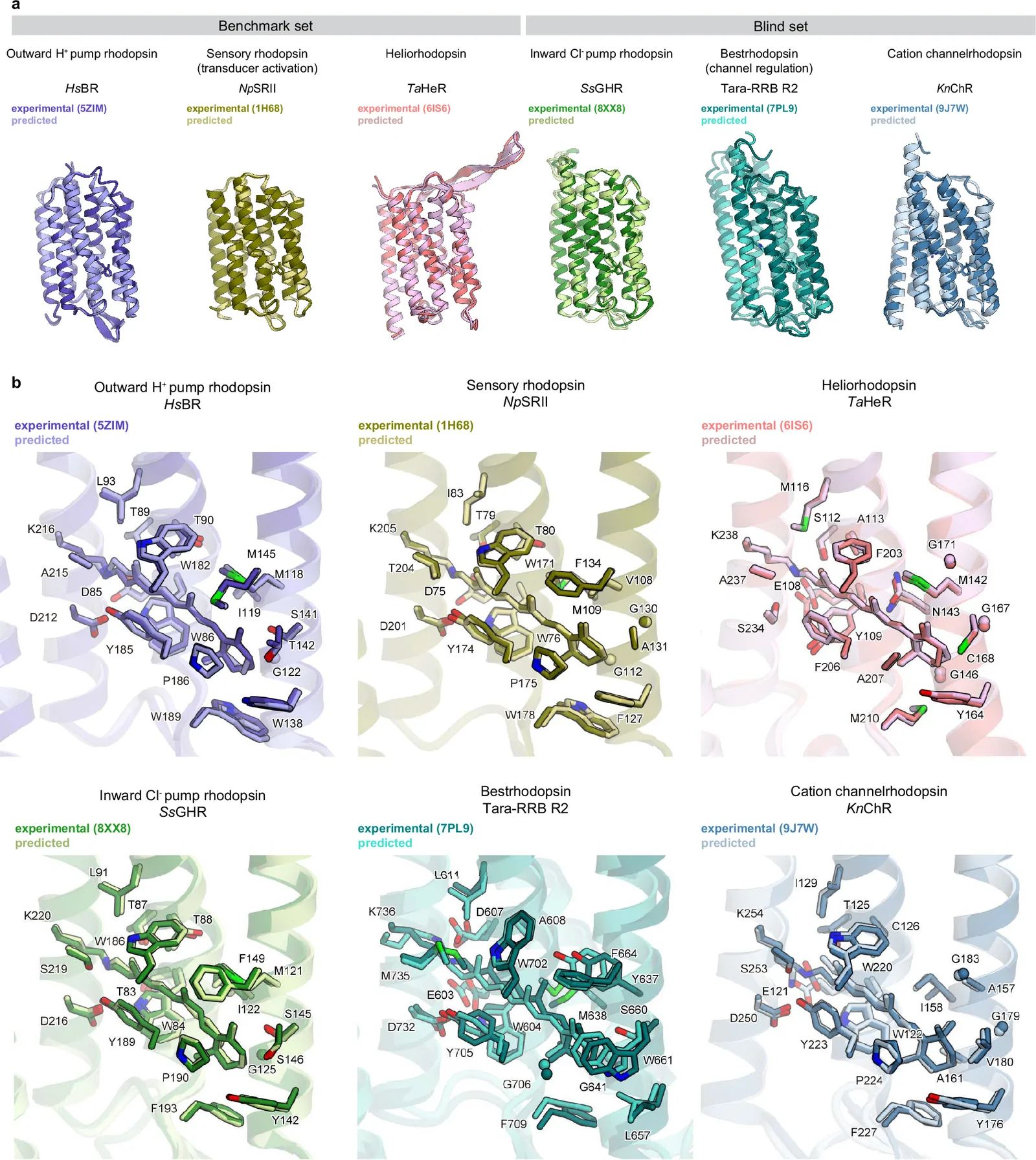
Accuracy of the predicted models. **a** Structural superposition between experimental (solid colors) and predicted (light colors) structures of representative rhodopsins: from the benchmark set, the outward proton pump rhodopsin *Hs*BR (PDB ID: 5ZIM), the transducer-activating rhodopsin *Np*SRII (PDB ID: 1H68), and the functionally uncharacterized heliorhodopsin *Ta*HeR (PDB ID: 6IS6); and from the blind set, the inward chloride pump rhodopsin *Ss*GHR (PDB ID: 8XX8), the channel-regulating rhodopsin Tara-RRB (PDB ID: 7PL9), and the cation-conducting channelrhodopsin *Kn*ChR (PDB ID: 9J7W). **b** Detailed view of the retinal-binding pockets. Transmembrane segments are depicted as translucent ribbons and retinal and protein side chains as solid sticks. TM 5, 6, and 7 are omitted for clarity.

To illustrate the accuracy of the predicted models, we show experimental and predicted structures for six representative rhodopsins spanning different functional classes (Fig. 2). In all cases, the overall fold was accurately reproduced, with loops largely correctly positioned (Fig. 2a). The retinal-binding pocket was also consistently modeled at sub-angstrom precision (Fig. 2b), including accurate handling of unusual 6-s-*cis* β-ionone ring conformations (as observed in the cation channelrhodopsin *Kn*ChR), consistent with the non-G rule^27–29^. These results show that current structure prediction methods reliably capture both the global microbial rhodopsin fold and the details of the retinal-binding pocket, supporting the inclusion of predicted structures to increase the diversity of our dataset.

### Overall structural conservation of the microbial rhodopsin fold

**Fig. 3:**
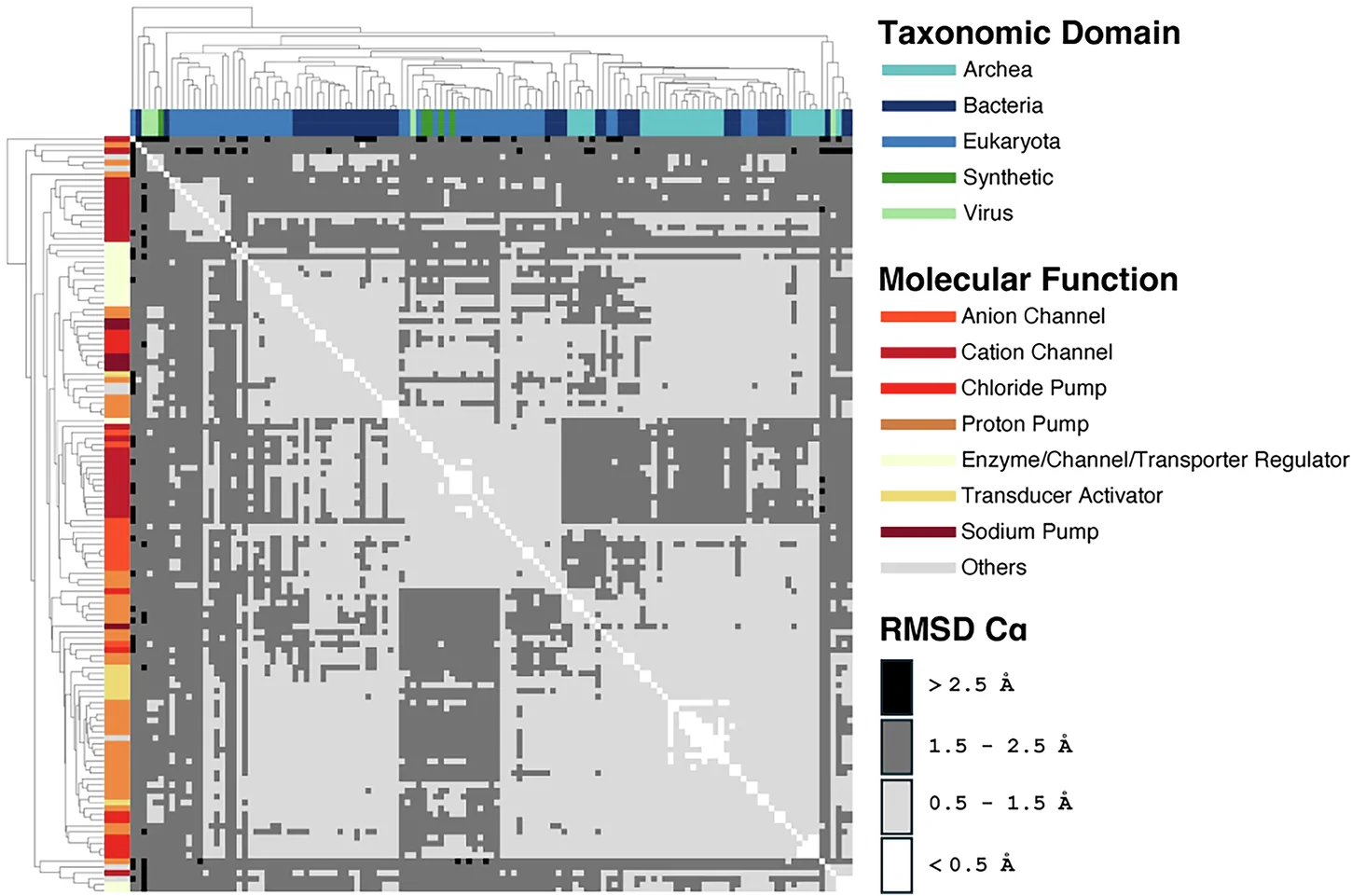
Structural landscape of microbial rhodopsins. The heatmap displays pairwise RMSD Cα values for all 129 dataset members. Rhodopsins are sorted according to hierarchical clustering based on structural similarity. Annotation bars display taxonomic domain (x-axis; green/blue) and molecular function (y-axis; orange/red). The greyscale bar indicates the RMSD values (light gray = 0.5–1.5 Å, dark gray = 1.5–2.5 Å, black ≥ 2.5 Å).

To analyze structural conservation across the dataset, we calculated pairwise RMSDs between Cα atoms of the transmembrane helices for all structure pairs. The resulting RMSD matrix (Fig. 3) reveals substantial structural conservation across the superfamily. However, sequence identity is low, averaging 21 ± 9% in the TM bundle (min = 7%) and 19 ± 9% in the full sequence (min = 6%). Hierarchical clustering of the RMSD matrix identified a single main structural cluster, confirming that the microbial rhodopsin fold is highly conserved across the superfamily. A generic residue-numbering system for microbial rhodopsins should therefore be based primarily on structural features rather than sequence.

### Development of the generic residue-numbering system

**Fig. 4:**
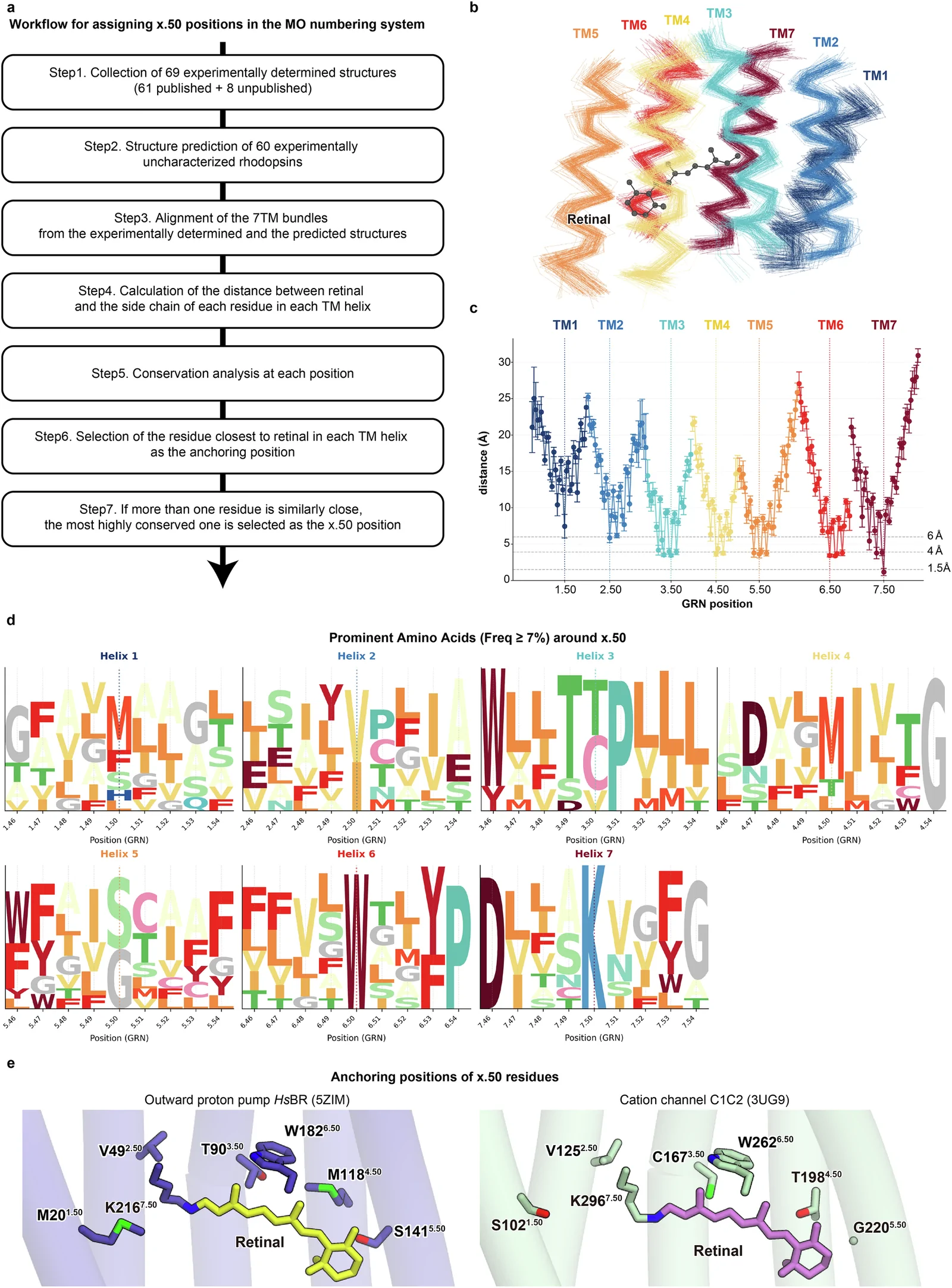
MO numbering anchor positions. **a** Workflow for determining the anchor position in each helix of microbial rhodopsins. **b** Structural alignment of the 129 dataset members used to select the consensus residues closest to retinal in each helix. **c** Average minimum distance from each structurally equivalent residue position to the retinal chromophore calculated across the dataset (closest side-chain atom, excluding hydrogen; an analogous graph for Cα distances is shown in Supplementary Fig. 2). Vertical lines indicate the defined X.50 anchor positions for each helix (1.50–7.50). Horizontal lines indicate key interaction distances: 1.5 Å approximates a covalent bond length and is relevant for the Schiff base linkage; 4 Å represents a typical cutoff for non-covalent interactions, delineating the direct binding pocket; and 6 Å encompasses the broader binding-site environment and is the radius used for iRMSD calculations. **d** Sequence logos depicting the conservation around the anchor positions in TM1–TM7 for the rhodopsins in the structural dataset (*n* = 129). **e** X.50 residues mapped onto representative structures of the outward proton-pumping rhodopsin *Hs*BR (left) and the cation-conducting channelrhodopsin C1C2 (right).

The MO numbering system is inspired by the Ballesteros–Weinstein (BW) system for G protein-coupled receptors but adapted to the unique architecture of microbial rhodopsins. As in the BW system, MO numbering codes consist of two numbers separated by a dot: the first denotes the helix (1–7), and the second denotes the position within the helix relative to an anchor residue defined as position 50. Residue numbers decrease toward the N-terminus and increase toward the C-terminus. For example, position 7.46 denotes a residue in TM7, four residues before the Schiff base-forming lysine (defined as 7.50). For residues in the N-terminus, extracellular loops, intracellular loops, or C-terminus, N-ter, ECL1-3, ICL1-3, or C-ter is used as the superscript, respectively. To relate generic positions to specific proteins, the sequence-specific residue number can be combined with the generic identifier, following the BW convention: for instance, D85^3.45^ refers to Asp85 located at generic position 3.45. This notation allows readers to identify both the absolute position in a given protein and its structural equivalent across the superfamily. Unlike the BW system, in which anchor residues are defined primarily by sequence conservation, the MO numbering system defines anchors primarily based on structural proximity to the retinal chromophore, reflecting the structure-based rationale of the approach. When multiple residues showed comparable proximity to retinal, sequence conservation and prior functional knowledge were used as additional criteria to select the final anchor position. This hierarchical, chromophore-centered strategy is summarized in Fig. 4a,b.

The distance profiles along each helix (Fig. 4c) exhibit the expected systematic variations, reaching minima near the central region of each helix and increasing toward the N- and C-terminal ends. The binding pocket (<4 Å around retinal) is formed by residues from TM3-7. Only two residues in TM2 are part of the extended pocket (<6 Å), while TM1 is at a peripheral location within the 7-TM bundle and further from retinal. The selected anchor residue in each helix is indicated by a vertical line in Fig. 4c. Sequence variability around the anchor positions (Fig. 4d) shows that they lie within or adjacent to regions of elevated sequence conservation despite the overall sequence divergence of the superfamily. Together, these observations support a chromophore-centered numbering strategy that is primarily guided by retinal proximity and refined by sequence conservation and prior functional knowledge, thereby capturing structurally and functionally critical positions across diverse microbial rhodopsins (Fig. 4e).

### Application of the generic residue-numbering system

**Fig. 5:**
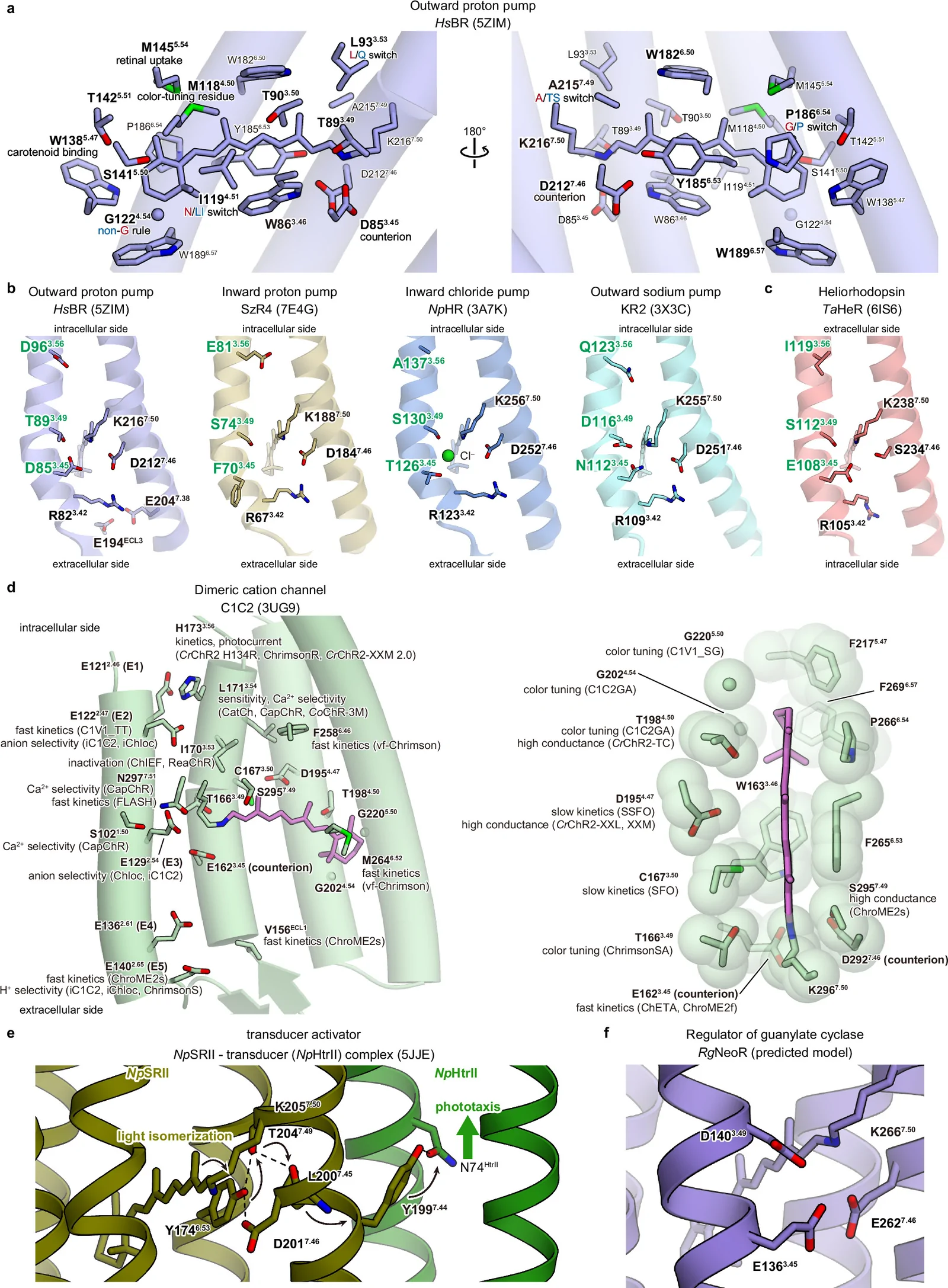
Demonstration of the MO numbering system. **a** Retinal-binding pocket of the outward proton pump *Hs*BR (PDB ID: 5ZIM), shown from two different orientations. Residues constituting the retinal-binding pocket are depicted as sticks. Bold labels indicate residues positioned on the front side of the retinal chromophore. Annotations below each label indicate the functional role of each residue. Blue and red annotations denote shifts toward shorter and longer wavelengths, respectively. **b** Schiff-base vicinity of representative ion-pumping rhodopsins: the outward proton pump *Hs*BR (PDB ID: 5ZIM), the inward proton pump SzR4 (PDB ID: 7E4G), the inward chloride pump *Np*HR (PDB ID: 3A7K), and the outward sodium pump KR2 (PDB ID: 3X3C) (left to right). Only TM3 and TM7 are shown as ribbons for clarity. The residues displayed correspond to functionally important positions. **c** Schiff-base vicinity of the heliorhodopsin *Ta*HeR (PDB ID: 6IS6). In (**b**, **c**), the residues of the DTD motif are highlighted in green. **d** Distribution of functionally important residues identified across various dimeric channelrhodopsin variants, mapped onto the overall structure (left) and the retinal-binding pocket (right) of C1C2 (PDB ID: 3UG9). Variant names associated with each mapped residue are shown adjacent to the corresponding positions. Annotations below each label indicate the functional properties or engineered mutations reported for that variant. **e** Transducer-activating rhodopsin *Np*SRII (khaki) and its cognate transducer *Np*HtrII (green) from the archaeon *Natronomonas pharaonis* (PDB ID: 5JJE). Residues critical for signal activation are shown in stick representation. TM1 is omitted for clarity. **f** Three key residues implicated in long-wavelength absorption in the guanylate cyclase–fused rhodopsin *Rg*NeoR. The structure shown is a model predicted using Boltz-1.

To demonstrate the application of the MO numbering system, we first focus on residues that form the retinal-binding pocket, using *Hs*BR as a reference (Fig. 5a). Retinal is covalently bound to K216^7.50^ (GRNs are shown as superscripts) via a Schiff base linkage and is surrounded by 18 additional residues from TM3-7 (5 from TM3, 3 from TM4, 4 from TM5, 4 from TM6, and 2 from TM7). Two highly conserved carboxylates, D^3.45^ and D^7.46^, located near K^7.50^ (D85^3.45^ and D212^7.46^ in *Hs*BR), are historically referred to as the “Schiff base counterions” and stabilize the positive charge of the protonated Schiff base. Residues at positions 3.53, 4.51, 6.54, and 7.49—known as the L/Q, N/LI, G/P, and A/TS switches, respectively—contribute to spectral tuning in microbial rhodopsins^30–33^. In addition, position 4.54 (G122^4.54^ in *Hs*BR) plays an important role in determining retinal planarity: when a non-glycine residue occupies this position, steric clash with the retinal β-ionone ring can induce rotation and favor a 6-s-*cis* retinal conformation (the “non-G rule”)^27–29^. Two other notable positions near the β-ionone ring are 5.54 and 5.47. Although retinal is enclosed by TM3-7, many microbial rhodopsins have a small lateral opening between TM5 and TM6 that connects the retinal pocket to the lipid bilayer^13,15,34–36^. The shape of this opening varies among microbial rhodopsins: in some bacterial ion-pumping rhodopsins, residue 5.47 contributes to its formation, whereas in heliorhodopsins (HeR) both 5.54 and 5.47 contribute. Functionally, 5.54 has been implicated in retinal uptake—the G171^5.54^W mutation in *Ta*HeR inhibits this process^15^—while 5.47 is involved in binding the carotenoid antenna pigment, as evidenced by the G178^5.47^W mutation in *Gloeobacter* rhodopsin, which inhibits carotenoid binding^37,38^.

The MO numbering system also facilitates comparative analyses across subfamilies. As an example, in ion-pumping rhodopsins, the identity of the transported substrate and the direction of transport are primarily influenced by three residues at positions 3.45, 3.49, and 3.56, which are located on one face of TM3 (Fig. 5b)^2,4,25^. Ion-pumping rhodopsins containing D^3.45^, T^3.49^, and D^3.56^—known as the DTD motif—predominantly function as outward proton pumps. Upon photoactivation, the proton is released from the Schiff base to the extracellular bulk solvent via T^3.49^, D^3.45^, and the proton release group composed of E^ECL3^ and E^7.38^, accompanied by a conformational change of the key arginine residue R^3.42^. A new proton is subsequently supplied from the intracellular bulk solvent to the Schiff base via D^3.56^, thereby accomplishing net transport of a single proton from the intracellular to the extracellular side. In contrast, inward proton-pumping rhodopsins, which comprise schizorhodopsins (SzRs) and xenorhodopsins (XeRs), lack one of the two counterions near the Schiff base. Specifically, D85^3.45^ in *Hs*BR is replaced by F^3.45^ in SzRs, whereas D212^7.46^ in *Hs*BR is replaced by P^7.46^ in XeRs. The D^3.45^–T^3.49^–D^3.56^ motif is replaced by FSE in SzRs and by DSA, DTL, or DTS in XeRs, respectively, and the proton release group conserved in outward proton pumps is absent in both SzRs and XeRs^39,40^. Inward chloride pumps have T^3.45^-S^3.49^-A/D^3.56^ or N^3.45^-T^3.49^-Q^3.56^ motifs, and the loss of negative charge caused by replacement of D^3.45^ with T^3.45^ or N^3.45^ is compensated by binding of the chloride substrate^41–43^. Outward sodium pumps share the N^3.45^-D^3.49^-Q^3.56^ motif, in which transient proton transfer from the Schiff base to D^3.49^ enables the sodium ion to pass through the Schiff base region^24,35,44,45^.

The MO numbering system is also applicable to rhodopsins with less canonical topologies or accessory structural features. For instance, Heliorhodopsins (HeRs) have a canonical overall fold but an inverted topology, with an intracellular N-terminus, an extracellular C-terminus, and distinctive extracellular loops (Fig. 5c). Despite this inversion, our structural alignment and annotation approach remains effective. At the sequence level, HeRs retain a TM3 counterion at position 3.45, whereas the TM7 counterion at 7.46 is replaced by serine. While the molecular functions of most HeRs remain unclear, a recent study suggested that V2HeR3 functions as a proton transporter^46^, and the MO numbering system may facilitate future comparative analyses as more functional data become available.

Channelrhodopsins, widely used in optogenetics, present a challenge for systematic comparison: mutagenesis and protein engineering have generated numerous functional variants, while metagenomics continues to uncover natural diversity. The MO numbering system enables mapping of this diversity onto a common structural framework, as illustrated for the C1C2 structure (Fig. 5d). Unlike ion-pumping rhodopsins, the TM3 residues at positions 3.45, 3.49, and 3.56 are not essential for channel function in canonical dimeric channelrhodopsins; substitutions at these and nearby positions, such as E^3.45^T or H^3.56^R, primarily affect channel kinetics^47,48^. Instead, ion selectivity and kinetics are largely determined by five glutamates (E1–E5, corresponding to E^2.46^, E^2.47^, E^2.54^, E^2.61^, and E^2.65^) aligned along TM2^49–52^. The DC gate, formed by D^4.47^ and C^3.50^, controls channel closure kinetics and light sensitivity, enabling bistable optical control of neuronal activity^53–55^. Figure 5d summarizes additional positions implicated in spectral tuning, conductance, and kinetics^27,56–68^, and Supplementary Fig. 3 summarizes positions that affect properties in trimeric channelrhodopsins^16,28,69–75^.

Transducer-activating rhodopsins (sensory rhodopsins) relay light signals to downstream effectors. Structural, spectroscopic, and mutational studies have clarified the activation mechanism of *Np*SRII^76–78^ (Fig. 5e). *Np*SRII forms a 2:2 complex with its transducer HtrII through an interface formed by TM6 and TM7 of *Np*SRII and TM1 and TM2 of HtrII. In *Np*SRII, retinal photoisomerization induces structural changes in residues Y174^6.53^, L200^7.45^, D201^7.46^, and T204^7.49^, which are transmitted to HtrII via the interaction between Y199^7.44^ and N74 of HtrII^78,79^. Notably, the key residue T204^7.49^ in *Np*SRII is not conserved in *H. salinarum* SRI (*Hs*SRI), suggesting that this signaling pathway may be specific to *Np*SRII rather than a general feature of transducer-activating rhodopsins^80^.

Enzyme-fused rhodopsins, such as rhodopsin-guanylyl cyclases and rhodopsin-phosphodiesterases, couple light detection to enzymatic activity. A characteristic feature of these proteins—shared with some channel-fused rhodopsins—is an additional TM0 helix at the N-terminus of the seven-transmembrane core^6,10^. The MO numbering system annotates the seven helices forming the retinal-binding domain, excluding TM0. For NeoR, the longest-wavelength rhodopsin reported to date, functional studies have identified three acidic residues essential for activity: E136^3.45^, D140^3.49^, and E262^7.46^ ^81^ (Fig. 5f). As structural and spectroscopic data on enzyme-fused rhodopsins accumulate, the MO numbering system will facilitate comparative analysis of their activation mechanisms.

The MO numbering system also accommodates microbial rhodopsins that exhibit gaps or insertions arising from local structural distortions (Supplementary Fig. 4). For example, ChRmine, a trimeric cation-conducting channelrhodopsin^82^, contains a constricted 3_10_ helical turn within TM6 that results in a gap in the sequence alignment^16^ (Supplementary. Fig. 4, salmon). In this case, the “missing” position (6.48) is simply skipped. Conversely, TaraRRB-R1, a channel-regulating rhodopsin^6^, contains a bulged π-helical turn in TM5 that introduces an additional residue between 5.45 and 5.46 (Supplementary. Fig. 4, green), which is designated 5.451.

Together, these applications demonstrate that the MO numbering system provides a unified framework for comparing rhodopsins across functional classes, taxonomic origins, and structural variations.

### Application of the MO numbering system to a large dataset of microbial rhodopsins

**Fig. 6:**
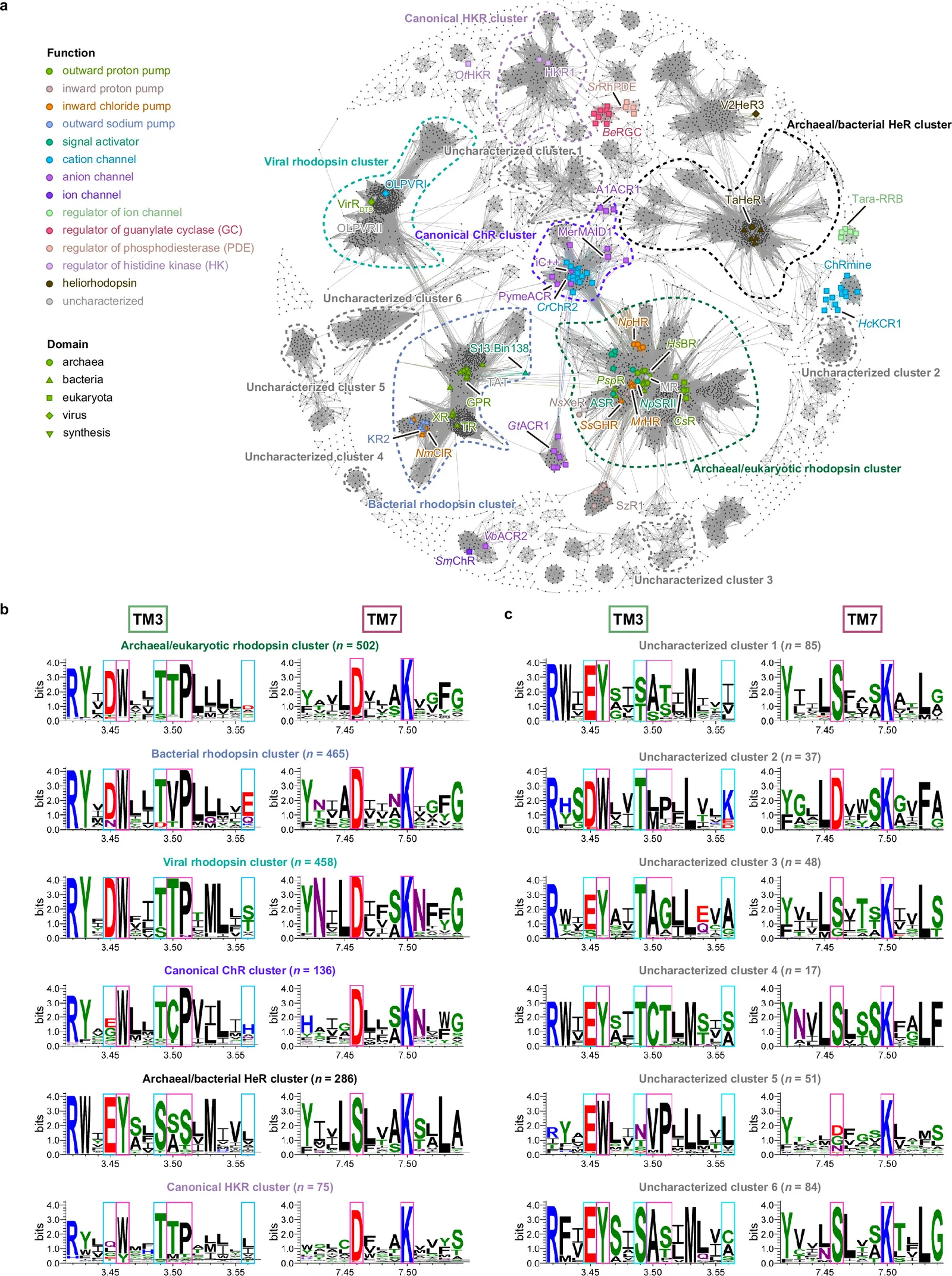
Application of the MO numbering system to a large dataset of microbial rhodopsins. **a** Sequence similarity network composed of approximately 40,000 non-redundant microbial rhodopsin sequences, including a large collection of uncharacterized rhodopsin-like sequences. Six major clusters containing functionally characterized rhodopsins and six of the 17 clusters containing only uncharacterized rhodopsins are highlighted with dashed circles. Representative rhodopsins are mapped onto the clusters, and the colors and shapes of the labels indicate function and domain, respectively. **b** Sequence logos of TM3 and TM7 for the six major clusters containing functionally characterized rhodopsins. **c** Sequence logos of TM3 and TM7 for six of the 17 clusters containing only uncharacterized rhodopsins. In (**b**, **c**), cyan rectangles indicate residues of the DTD motif and magenta rectangles indicate highly conserved residues within each cluster.

To test the MO numbering system at scale, we applied it to a large sequence dataset that included both characterized and uncharacterized rhodopsins. We combined the 129 functionally characterized rhodopsins in our curated dataset with a large collection of uncharacterized rhodopsin-like sequences, yielding an initial set of more than 300,000 sequences from archaea, bacteria, eukaryotes, and viruses. After curation, we obtained approximately 40,000 non-redundant sequences. From pairwise sequence similarities, we constructed a protein similarity network in which nodes represent individual sequences and edges connect pairs exceeding a defined similarity threshold (Fig. 6a). The network resolved 14 clusters containing functionally characterized rhodopsins and 17 clusters containing only uncharacterized rhodopsins (Supplementary Fig. 5). The 14 characterized clusters comprise six major groups—archaeal/eukaryotic, bacterial, viral rhodopsins (VirRs)^83,84^, canonical channelrhodopsins (ChRs), canonical histidine kinase-fused rhodopsins (HKRs)^85,86^, and canonical heliorhodopsins (HeRs)^9^—and eight minor groups: schizorhodopsins (SzRs)^87^, cryptophyte anion-conducting channelrhodopsins (ACRs)^88^, alveolate ChRs^23^, pump-like channelrhodopsins (PLCRs)^89^, guanylate cyclase-/phosphodiesterase-fused rhodopsins (RhoGC/RhoPDEs)^10^, bestrophin-fused rhodopsins (BestRs)^6^, noncanonical HKRs^90^, and eukaryotic/viral HeRs^46^ (Supplementary Fig. 5).

To validate the clustering and explore uncharacterized rhodopsins, we predicted structures for 11 sequences from the periphery of 5 of the 14 functionally characterized clusters, along with one representative member from each of the 17 uncharacterized clusters (Supplementary Fig. 6a). In total, we generated structural models for 28 rhodopsin-like proteins (Supplementary Fig. 6b). All 11 characterized and 13 of the 17 uncharacterized sequences (uncharacterized clusters 1–13) adopted a rhodopsin fold and were retained for further analysis. Of the 13 uncharacterized rhodopsins, two lacked the conserved Lys^7.50^ and one exhibited an incorrectly predicted retinal-binding pose. Several uncharacterized clusters exhibited distinctive structural features: for example, the rhodopsin in cluster 2 has an unusually long TM1, whereas the rhodopsin in cluster 10 contains β-strands in ECL2 and ECL3 that form an extracellular β-sheet (Supplementary Fig. 6b).

To examine sequence conservation, we generated sequence logos in selected clusters **(**Fig. 6b,c**;** Supplementary Figs. 7- 9). Conserved residues grouped near the X.50 anchor positions at approximately four-residue intervals, consistent with enrichment on the retinal-facing side of each helix; lipid-exposed positions showed lower conservation. Across all clusters, residues in TM3, TM6, and TM7 were more conserved than those in other helices. The arginine at position 3.42 is highly conserved across all functionally characterized clusters—with the exception of the PLCR cluster—consistent with its known key functional roles^91–95^. Residues forming the retinal-binding pocket (positions 3.46, 6.50, 6.53, and 6.54) and the Schiff base counterions (positions 3.45 and 7.46) are well conserved within characterized clusters; however, position 7.46 is more conserved than 3.45. Most characterized clusters have a highly conserved Asp at 7.46, but the alveolata ChR, bacterial/archaeal HeR, and eukaryotic/viral HeR clusters show highly conserved Asn, Ser, and Ser, respectively. Positions 3.50 and 3.51 vary markedly between characterized clusters—while remaining conserved within each—suggesting that these positions are linked to cluster-specific functions and serve as characteristic sequence signatures for these rhodopsin families. Similar conservation patterns were observed in uncharacterized clusters, although position 3.50 in cluster 2 and position 7.46 in cluster 5 showed reduced conservation (Fig. 6c and Supplementary Fig. 9).

To further characterize the uncharacterized clusters, we examined the sequence logos of clusters 1–6 together with their representative predicted structures, paying particular attention to residues surrounding the retinal Schiff base (Supplementary Figs. 9 and 10**)**. Although the overall sequence similarity to characterized rhodopsins was low, clusters 1, 3, 4, and 6 showed strong conservation of several residues, including H^1.50^, H^2.58^, E^3.45^, and S^7.46^ (Supplementary Fig. 9). Their representative predicted structures also exhibited an extended β-sheet between TM1 and TM2 (Supplementary Fig. 6b) and a positively charged intracellular surface (Supplementary Fig. 10b). These features are reminiscent of characterized HeRs, in which H^1.50^, H^2.58^, E^3.45^, and S^7.46^ have been implicated in photocycle regulation^9^. Thus, despite their low overall sequence similarity to canonical HeRs, clusters 1, 3, 4, and 6 likely represent distantly related heliorhodopsin-like families. Cluster 2 showed conservation of D^3.45^ and T^3.49^ in TM3 and D^7.46^ in TM7, whereas K was predominant at position 3.56. This combination suggests that cluster 2 may represent a previously unrecognized rhodopsin subfamily, potentially related to outward proton pumping or cation conductance. In contrast, cluster 5 did not exhibit sequence or structural signatures characteristic of functionally characterized microbial rhodopsins. Moreover, this cluster lacked a conserved residue corresponding to the canonical Schiff base counterion at position 7.46. These features suggest that cluster 5 may represent a rhodopsin family with unusual photochemical or functional properties, warranting future experimental characterization.

## Discussion

The repertoire of microbial rhodopsins continues to expand. With estimates of global microbial diversity ranging from 10^6^ ^96^ to 10^12^ species^97^, and over two million bacterial and archaeal genomes now sequenced^98^, the number of identified rhodopsin genes—already exceeding 10,000^99^—will undoubtedly grow further. This expanding diversity, combined with ongoing optogenetics engineering efforts, has made a unified residue-numbering system increasingly necessary.

The limitations of existing numbering approaches become apparent when comparing across functional classes. Ion-pumping rhodopsins have historically been described using BR-based residue numbering, whereas channelrhodopsins are typically numbered according to ChR2- or C1C2-based systems. The three-residue TM3 motif introduced in 2013-2014 provides a useful signature for classifying ion-pump functions^24,25^; however, in channelrhodopsins, functional properties are determined primarily by other residues, including five glutamates in TM2, rather than by the TM3 motif. These differences underscore the need for a broadly applicable numbering system, which motivated the development of the MO numbering system.

The sequence similarity network provides insight into the evolutionary relationships among microbial rhodopsins (Fig. 6a and Supplementary Fig. 5). Rhodopsins from archaea and eukaryotes form a single major cluster (the archaeal/eukaryotic rhodopsin cluster), whereas bacterial rhodopsins occupy a distinct cluster (the bacterial rhodopsin cluster). This pattern indicates that eukaryotic microbial rhodopsins are more closely related to archaeal than to bacterial rhodopsins, consistent with previous phylogenetic and structural analyses^100–103^.

Rhodopsins within the same cluster but with different functions likely diverged from a common ancestor, whereas functionally similar rhodopsins in different clusters likely arose through convergent evolution. For example, both the archaeal/eukaryotic and bacterial clusters include chloride-pumping rhodopsins, yet their TM3 motifs differ (T^3.45^-S^3.49^-A/D^3.56^ versus N^3.45^-T^3.49^-Q^3.56^, respectively), suggesting that chloride-transport function evolved independently in these lineages. Within clusters, oligomeric assembly and sequence motifs are conserved despite functional diversification. This conservation likely explains why researchers have been able to interconvert functions through relatively few mutations, even among rhodopsins sharing only ~30% sequence identity^51,52,79,104–107^.

The conservation of oligomeric assembly within clusters raises a question: what determines oligomeric state? All characterized microbial rhodopsins form specific oligomers, ranging from dimers to hexamers, and rhodopsins within the same clade consistently adopt the same oligomeric state^11,12,15,16,87^. Yet residues on the outer, lipid-exposed surface are generally poorly conserved, even in helices that participate in oligomeric interfaces. This suggests that oligomeric state is not dictated by a fixed set of conserved interface residues. In some rhodopsins, such as *Np*HR, outer-facing residues do contribute to interprotomer contacts and stabilize oligomerization^108^, but these residues are not conserved across clusters, implying that they fine-tune oligomer stability within a family rather than specifying oligomeric state. Instead, we propose that the type of oligomer formed is largely determined by helix-packing geometry, specifically how the transmembrane helices are tilted and arranged relative to one another. Helix tilt can be influenced by glycine and proline residues within helices, as well as loop architecture. In particular, the length and orientation of the ECL1 have been proposed to distinguish trimeric from pentameric or hexameric assemblies^100^. However, pentameric rhodopsins such as GR and TR form trimers in DDM micelles^100,109^, suggesting that the ECL1 may bias helix tilt toward particular arrangements without rigidly fixing protomer number. This observation implies that the membrane or detergent environment also contributes to stabilizing a given assembly.

Beyond evolutionary and oligomeric insights, our study also assessed the utility of structure prediction for rhodopsin analysis. Structure prediction methods performed well for rhodopsins. Predictions captured the overall fold and the detailed architecture of the retinal-binding pocket, often correctly reproducing the retinal configuration and orientation, including whether the chromophore adopts an all-*trans* geometry or a 6-*s-cis* conformation consistent with the non-G rule^28,29,35^. Our backbone RMSDs (0.61 Å for the benchmark set and 0.80 Å for the blind set) are comparable to those reported for Boltz-1 in the Runs N’ Poses benchmark^110^ (0.56 Å) and lower than AlphaFold2 predictions of GPCRs^111^ (1.12–1.55 Å). The accuracy of retinal placement is particularly notable: our mean retinal RMSDs of 0.41 Å (benchmark set) and 0.62 Å (blind set) are substantially lower than the 2.30 Å mean ligand RMSD that Boltz-1 achieved on diverse protein-ligand complexes in the Runs N’ Poses benchmark. This likely reflects the strong structural constraints imposed by the retinal-binding pocket, including the covalent Schiff base linkage to the conserved lysine.

However, inaccuracies were frequently observed in fine structural details, including the placement of loop regions and side-chain rotamers. For example, in the predicted structure of *Kn*ChR, the retinal chromophore is not covalently linked to the Schiff base-forming lysine (K^7.50^) (Fig. 2b and Supplementary Fig. 11a). In *Gt*ACR1, the β-ionone ring of the retinal adopts an incorrectly twisted conformation^14^ (Supplementary Fig. 11b). In Tara-RRB R2, the cytoplasmic ends of TM0, TM1, TM2, and TM7 show marked deviations from the experimentally determined structure (Fig. 2a and Supplementary Fig. 11c). In *Hc*KCR2, the architecture of the potassium selectivity filter is incorrectly predicted^28^ (Supplementary Fig. 11d). These issues may become problematic when predicted structures are used e.g. as inputs for molecular dynamics simulations or for interpreting spectroscopic measurements, where accurate loop conformations and side-chain rotamers are critical. Thus, while current structure prediction methods are sufficiently accurate for comparative analyses and for capturing the overall architecture and retinal environment, they do not replace experimental structure determination when precise atomic detail or characterization of conformational dynamics is required.

The MO numbering system offers the microbial rhodopsin field a common vocabulary. As the repertoire of rhodopsins continues to expand through metagenomics and structural prediction, we anticipate that it will become a standard tool for comparing residues, interpreting functional data, and guiding optogenetic engineering.

## Methods

### Structural dataset

We assembled a comprehensive structural dataset of 129 microbial rhodopsins spanning all major functional classes—proton pumps, chloride pumps, sodium pumps, cation channels, anion channels, sensory rhodopsins, and enzyme rhodopsins—with representatives from archaea, bacteria, and eukaryotes. Experimentally determined structures were obtained from the Protein Data Bank, supplemented by eight recently solved channelrhodopsin structures (*n* = 69). The experimental structures were partitioned into two validation cohorts based on PDB deposition date relative to the Boltz-1^112^ training data cutoff (September 2021): a benchmark set (*n* = 42) potentially overlapping with training data, and a blind test set (*n* = 27) comprising structures deposited after the cutoff. For each experimentally characterized protein, we generated Boltz-1 predictions to enable systematic assessment of prediction accuracy. We additionally predicted structures for 60 functionally characterized rhodopsins lacking experimental structures. All analyses were performed on chain A, with retinal chromophores identified by residue name and validated by proximity (≤6.0 Å to protein atoms).

### Structure prediction and validation

For Boltz-1 predictions, input sequences were trimmed to transmembrane domain boundaries when exceeding 400 residues. Retinal was specified as all-trans-retinal without the aldehyde oxygen (SMILES code: CC=C(C)C=CC=C(C)C=CC1=C(CCCC1(C)C)C), allowing Boltz-1 to predict the covalent Schiff base linkage to the conserved lysine. Prediction accuracy was assessed by comparing each model to its experimental counterpart using the combinatorial extension alignment algorithm (CEalign)^113^ (window size = 8, maximum gap = 30) with a two-pass refinement procedure: an initial global alignment using all Cα atoms, followed by refinement restricted to residue pairs with inter-Cα distance ≤ 3 Å after the initial superposition to exclude flexible regions with large structural divergence. Accuracy was quantified using three metrics: backbone Cα RMSD (overall fold accuracy), binding pocket RMSD (Cα atoms within 6.0 Å of retinal; local accuracy in the chromophore environment), and ligand RMSD (mean closest-atom distance between experimental and predicted retinal coordinates).

### Structure Conservation Analysis

We first aligned the shared TM bundle of all members of our dataset. Pairwise structural similarities (calculated using CEalign) were computed as Cα RMSD across TM 1–7 to generate a symmetric distance matrix. Our structure analysis leverages the conserved microbial rhodopsin fold by using the closest residue in each helix (TM1-TM6) and the Schiff-base lysine in TM7 as anchors. We then performed hierarchical clustering. For each GRN position, two distance profiles were calculated: (i) side-chain distances, from heavy atoms to the nearest retinal atom, and (ii) Cα distances, from backbone Cα to the nearest retinal atom. Equivalent residue pairs for all pairwise alignments were stored to create the blueprint of the MO numbering system.

### Generic Residue-Numbering System

We developed the MO numbering system by assigning GRNs based on structural equivalence relative to a reference structure, 7BMH, selected based on its low mean RMSD to all other structures. In the MO numbering system, residues are numbered relative to the helix-specific anchors using the format <Helix>.<Position> (e.g., 3.49, 3.50, 3.51). After annotating the reference structure, we used the stored residue pair equivalences to assign GRNs to all other structures. The resulting preliminary MO numbering system was curated to correct alignment artifacts, including helix truncations and register shifts arising from the repetitive nature of α-helical structures. Then, the less conserved outer parts of the helices were annotated up to the end of the helices by determining helical residues based on phi-psi angles of the backbone. The non-helical loop regions and tails received systematic identifiers.

### Microbial rhodopsin sequence search

To construct Hidden Markov Models (HMMs) for rhodopsin sequence exploration, known rhodopsins and their homologs were collected from NCBI, the UniProt Archive^114^, and the Marine Microbial Eukaryote Transcriptome Sequencing Project (MMETSP)^115^. One collection (subAEV: 3,173 sequences) was constructed mainly from archaeal-, eukaryotic-, and viral-derived rhodopsins^116^. Another collection (subB: 2,275 sequences) was mainly composed of bacterial-derived rhodopsins. A third collection (subH: 720 sequences) was composed of heliorhodopsins and their homologs. Profile HMMs for the subAEV, subB, and subH collections were constructed from sequence alignments computed using MAFFT (v7.453)^117^ with the “--genafpair” and “--maxiterate 1000” options. Using these HMMs, we searched the UniParc database (as of November 2020), the marine eukaryotic reference catalog^118^, the 1000 Plant Transcriptomes Initiative dataset^119^, MMETSP, and 10,032 metagenomic assemblies derived from various environments. Rhodopsin homologs were identified using hmmsearch (HMMER v3.3)^120^ with an e-value threshold of <1e^-5^ and a length threshold of ≥174 amino acids, corresponding to the HMM length. We clustered 309,570 protein sequences using CD-HIT^121^ (v.4.8.1) at 90% global identity (-c 0.90, -G 1) without additional coverage constraints (no -aS/-aL), resulting in 41,136 non-redundant sequences.

### Network analysis visualization

We collected reference rhodopsins (*n* = 147) that were functionally and/or structurally characterized, along with the 41,136 non-redundant, uncharacterized rhodopsin sequences. Because enzymatic rhodopsins contain additional domains, the rhodopsin regions of both reference and uncharacterized sequences were extracted using aligned regions based on hmmsearch (HMMER v3.3)^120^ with the three HMMs (subAEV, subB, and subH). To further remove redundancy within the uncharacterized dataset, protein clustering was performed using MMseqs2 (ver. 15-6f452)^122^ at 60% identity and 50% coverage, with options “--min-seq-id 0.6 -c 0.5 --cluster-mode 2 --cov-mode 0”. Cluster representatives (*n* = 5168) were combined with the reference rhodopsins (total *n* = 5,315).

Normalized pairwise similarity within the resultant sequences was computed using CPAP (https://github.com/yosuken/cpap), which performed all-against-all BLASTp with options “-evalue 1e-2 -dbsize 100000000” and quantified normalized similarity scores between all possible protein pairs based on high-scoring segment pairs (HSPs) that have ≥20% identity and are ≥20 amino acids in length, as follows:

Let ${n}_{{AB},i}$ be the length-normalized bit score (i.e., bit score per position) of the HSP covering position i of protein A, where BLASTp is performed using A as the query and B as the subject. If multiple HSPs cover position i, the maximum value is used.

1 $${M}_{{AB},i}=\max ({n}_{{AB},i})$$

Let ${L}_{A}$ be the length of protein A. Let ${N}_{{AB}}$ $(0\le {N}_{{AB}}\le 1)$ be the normalized similarity score between proteins A and B.

2 $${U}_{{AB}}=\frac{{\sum }_{i}{M}_{{AB},i}}{{\sum }_{i}{M}_{{AA},i}},0\le i\le {L}_{A}$$

3 $${N}_{{AB}}=\frac{1}{2}\left({U}_{{AB}}+{U}_{{BA}}\right)$$

The resulting network was visualized using Gephi (ver. 0.10.1). Edges were drawn between protein pairs with normalized similarity scores ≥ 0.2.

### Sequence logo generation

Sequence logos were generated using WebLogo3^123^. Sequences were aligned according to their MO numbering positions, and logos were computed for each transmembrane helix. For cluster-specific analyses, sequences were grouped based on their assignment in the sequence similarity network, and separate logos were generated for each TM region (TM1-7). Amino acid frequencies are displayed as information content in bits.

### Molecular graphics

Molecular structures were rendered with CueMol (v2.3; http://cuemol.org), except Supplementary Fig. 4b, which was prepared with UCSF ChimeraX (v1.11)^124^. Figures were assembled and labeled in Adobe Illustrator 2026 (v30.6).

### Statistical analysis

Correlations between structural and sequence metrics were assessed using Pearson’s correlation coefficient with two-tailed tests of significance. Analyses were performed in Python 3.10.

## Supplementary information

Supplementary Information

## Data Availability

The structure analysis data is available at https://doi.org/10.5281/zenodo.18147121, under a Creative Commons Attribution 4.0 (CC-BY-4.0) licence. The cryo-EM density maps and atomic coordinates of the eight experimentally determined channelrhodopsin structures have been deposited in the Electron Microscopy Data Bank (EMDB) and the Protein Data Bank (PDB) under the following accession codes: bReaChES (PDB 44JM, EMDB EMD-82643), CoChR (PDB 44JN, EMDB EMD-82644), ChroME2s (PDB 44JO, EMDB EMD-82645), CnChR2 (PDB 44JP, EMDB EMD-82646), KnChR (PDB 44JQ, EMDB EMD-82647), TsChR (PDB 44JR, EMDB EMD-82648), R2ACR (PDB 44JS, EMDB EMD-82649), and A1ACR1 (PDB 44JT, EMDB EMD-82650).

## Code availability

Dataset management and CIF parsing were performed using ProtOS, an in-house data-management library (https://github.com/flurinh/protos). Structure coordinates, alignments, RMSD matrices, GRN tables and all analysis code are provided at https://github.com/flurinh/mogrn. We also provide a web-based annotation tool (available at https://rhodo.psi.ch/) where users can upload a sequence and receive the corresponding MO annotation. The tool also provides pre-calculated GRN tables for commonly studied rhodopsins. The code is released under the MIT License.
