# Supplementary material for: A generic residue-numbering system for microbial rhodopsins
Source: Npj Struct Biol. 2026 Nov 5;1(1):4. doi: 10.1038/s44511-026-00004-w (PMC13739731; doi:10.1038/s44511-026-00004-w)
Supplement: Supplementary file 1 [file 44511_2026_4_MOESM1_ESM.pdf]

Supplementary Figures

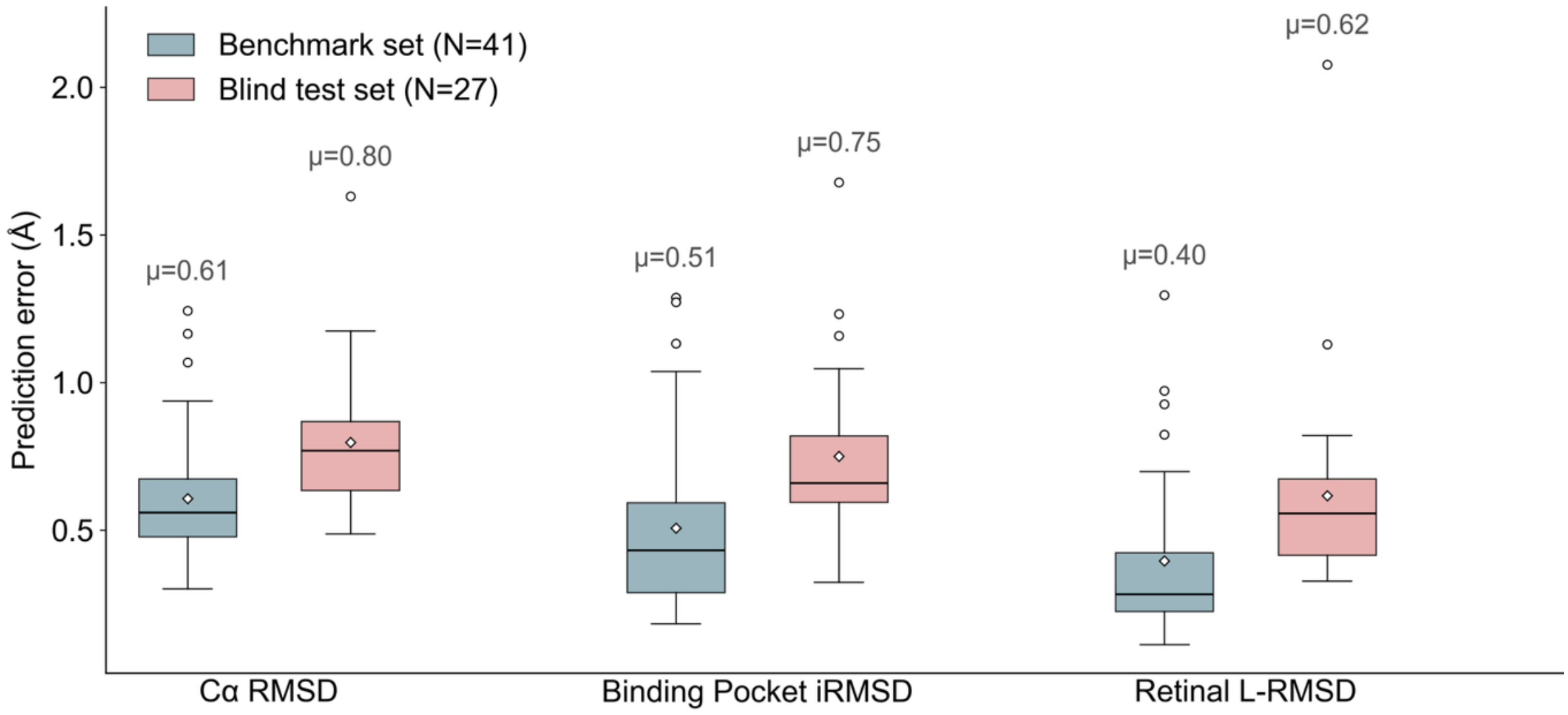

Supplementary Fig. 1 | Accuracy of the predicted models. Prediction errors for the two validation sets: benchmark set A (blue; n=42) and blind set B (red; n=27 for backbone, n=26 for pocket). The prediction errors are presented separately for the entire structure (global Cα RMSD), for the binding pocket (iRMSD), and for retinal (L-RMSD). Means are shown over each box. Only four predicted models in benchmark set A showed an error larger than 1 Å in the binding-pocket residues (iRMSD): TR (PDB ID: 5AZD); *Ns*XeR (PDB ID: 6EYU); Chrimson (PDB ID: 5ZIH), and XR (PDB ID: 3DDL). Four models in blind set B also exceeded this threshold: *Er*NaR (PDB ID: 8QLF) and three potassium channelrhodopsins (8H86, 8H87, and 8JXO). The most challenging cases were in blind set B: B1ChR2 (PDB ID: 8JXO, pocket iRMSD 1.68 Å, L-RMSD 2.08 Å) and Tara-RRB (PDB ID: 7PL9), a rhodopsin functionally coupled to the bestrophin chloride channel (excluded from pocket analysis).

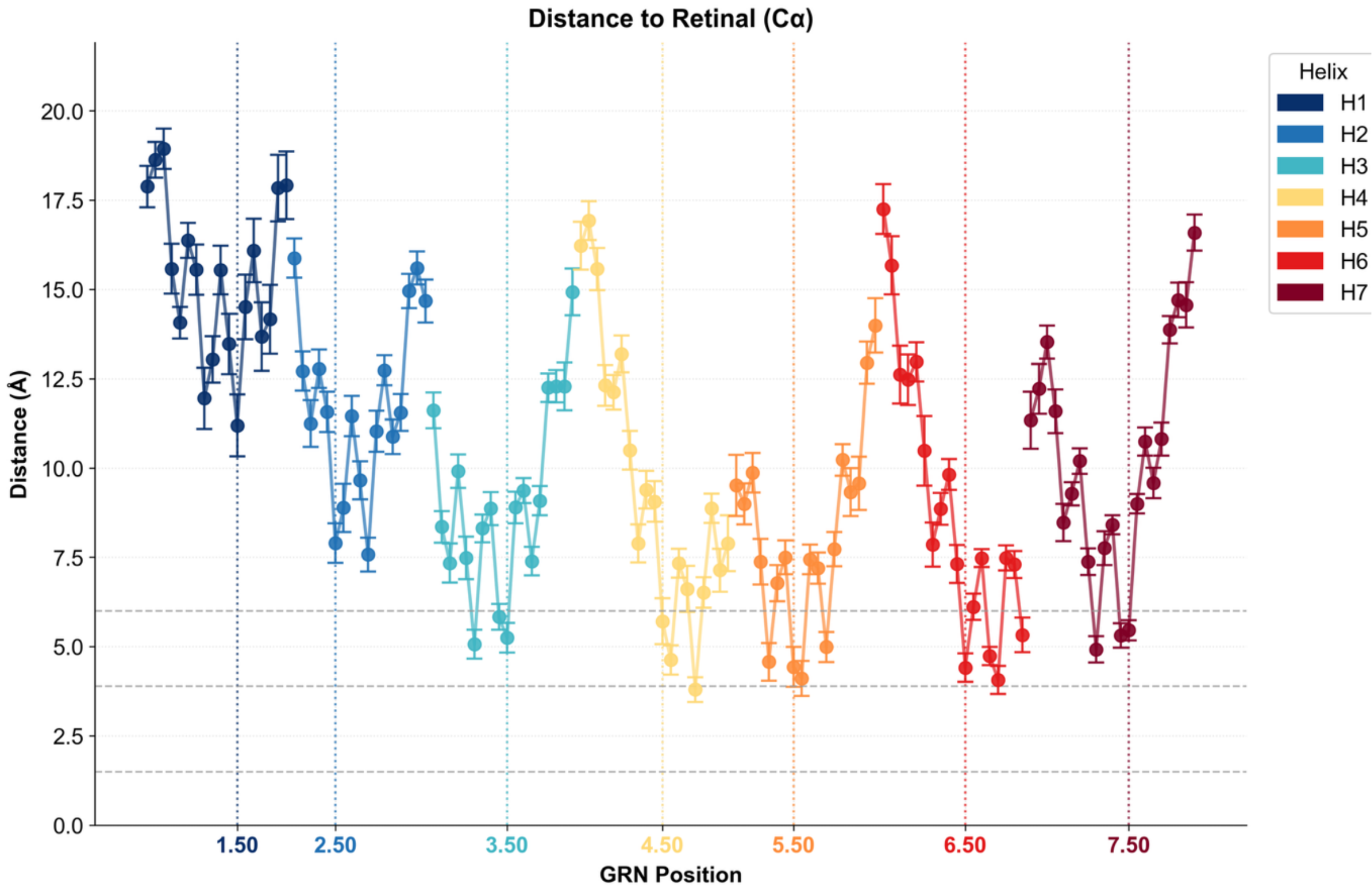

Supplementary Fig. 2 | Average minimum distance from each structurally equivalent residue position to the retinal chromophore (closest Cα atom), calculated across the dataset. Vertical lines indicate the defined X.50 anchor position for each helix (1.50–7.50). Horizontal lines indicate key interaction distances for side chains (shown for comparison with **Figure 4b**): 1.5 Å approximates a covalent bond length and is relevant for the Schiff base linkage; 4 Å represents a typical cutoff for non-covalent interactions, delineating the direct binding pocket; and 6 Å encompasses the broader binding-site environment and is the radius used for iRMSD calculations.

Trimeric potassium-selective channel
*Hc*KCR1 (8H86)

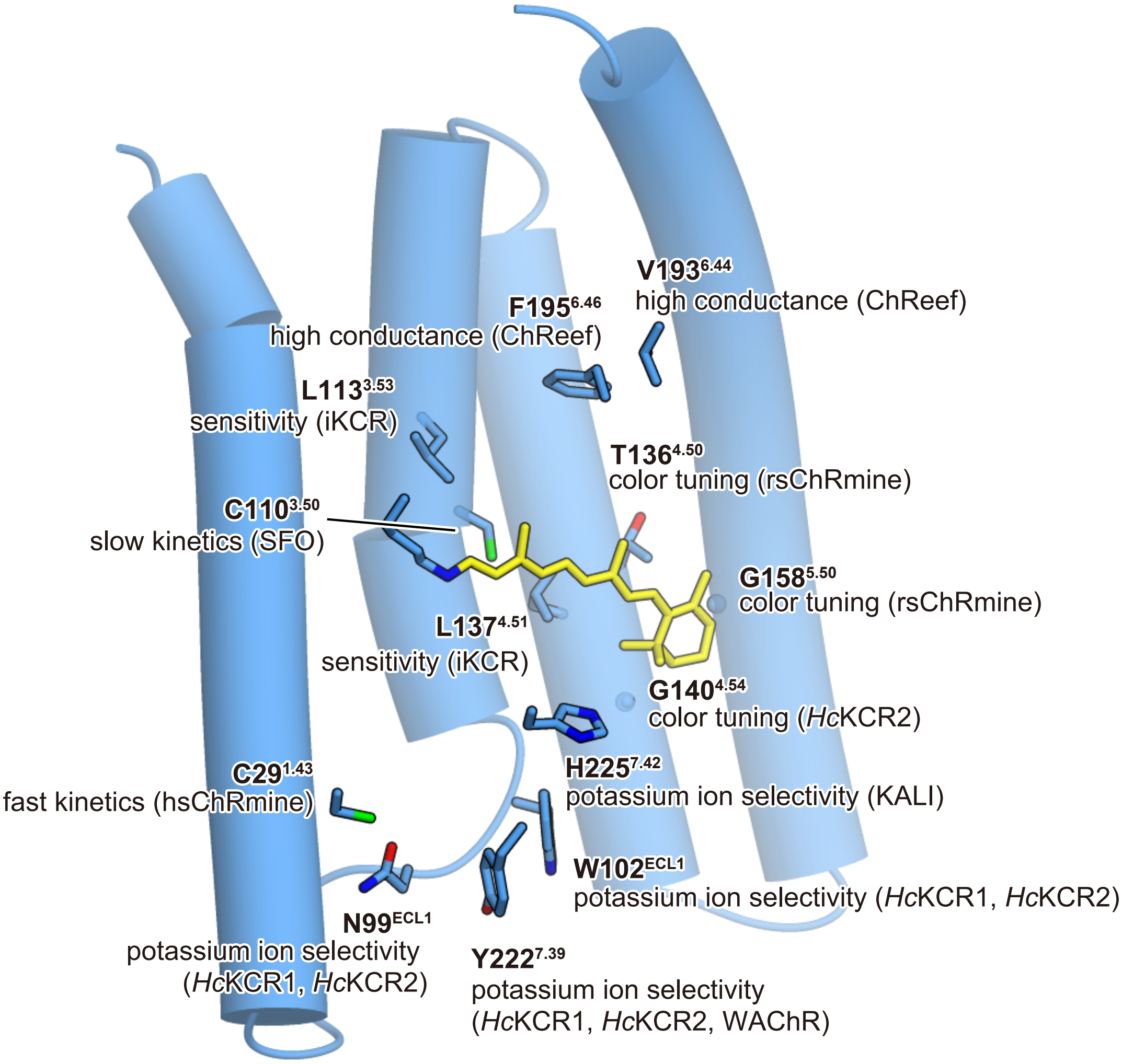

Supplementary Fig. 3 | Distribution of functionally important residues identified across various trimeric channelrhodopsin variants. Residues are mapped onto the overall structure of the potassium-selective channelrhodopsin *Hc*KCR1 (PDB ID: 8H86).

a

*Hs*BR_no	179	180	181	182	183	184	185	186	187
ChRmine_no	221	-	222	223	224	225	226	227	228
MO_no	6.47	6.48	6.49	6.50	6.51	6.52	6.53	6.54	6.55
*Hs*BR	V	V	L	W	S	A	Y	P	V
ChRmine	M	-	L	W	I	L	F	P	L

*Hs*BR_no	135	136	-	137	138	139	140	141	142
TaraRRB_no	250	251	252	253	254	255	256	257	258
MO_no	5.44	5.45	5.451	5.46	5.47	5.48	5.49	5.50	5.51
*Hs*BR	F	V	-	W	W	A	I	S	T
TaraRRB R1	Y	A	I	F	L	S	A	S	W

b

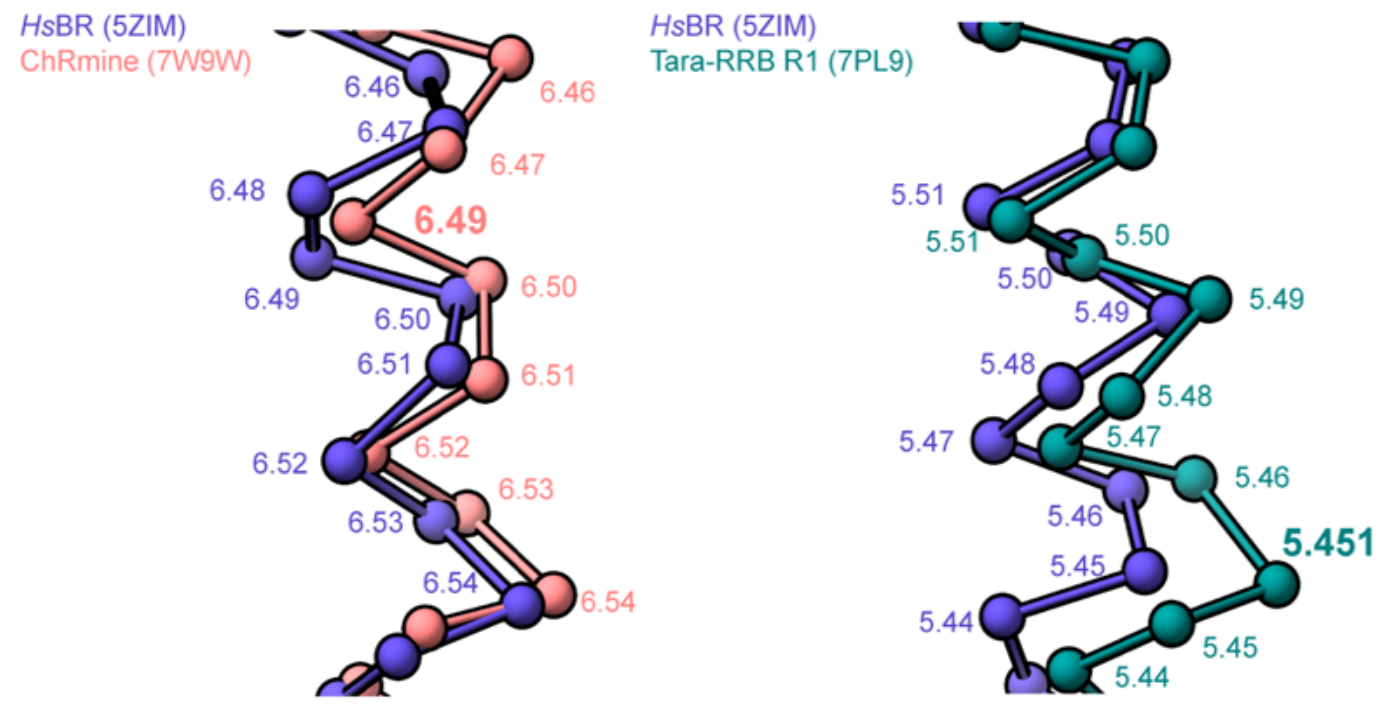

Supplementary Fig. 4 | Rules for insertions and gaps in the MO numbering system. a Structure-based alignment of TM6 between the reference *HsBR* and ChRmine (top) and of TM5 between *Hs*BR and TaraRRB-R1 (bottom). *Hs*BR_no and ChRmine_no/TaraRRB_no indicate the residue numbers in each protein, and the MO numbering row shows the corresponding numbers in the MO numbering system. In the gap in the ChRmine sequence, the GRN is skipped. The insertion in TaraRRB-R1 is assigned a fractional GRN (5.451). **b** Superposition of Cα backbone traces of the reference *Hs*BR (PDB ID: 5ZIM; blue) with ChRmine (PDB ID: 7W9W; pink, left) and with TaraRRB-R1 (PDB ID: 7PL9; green, right). Numbers along the helices indicate the GRNs, highlighting the positions of the gap (6.49, left) and the fractional GRN (5.451, right) corresponding to the alignment shown in panel a.

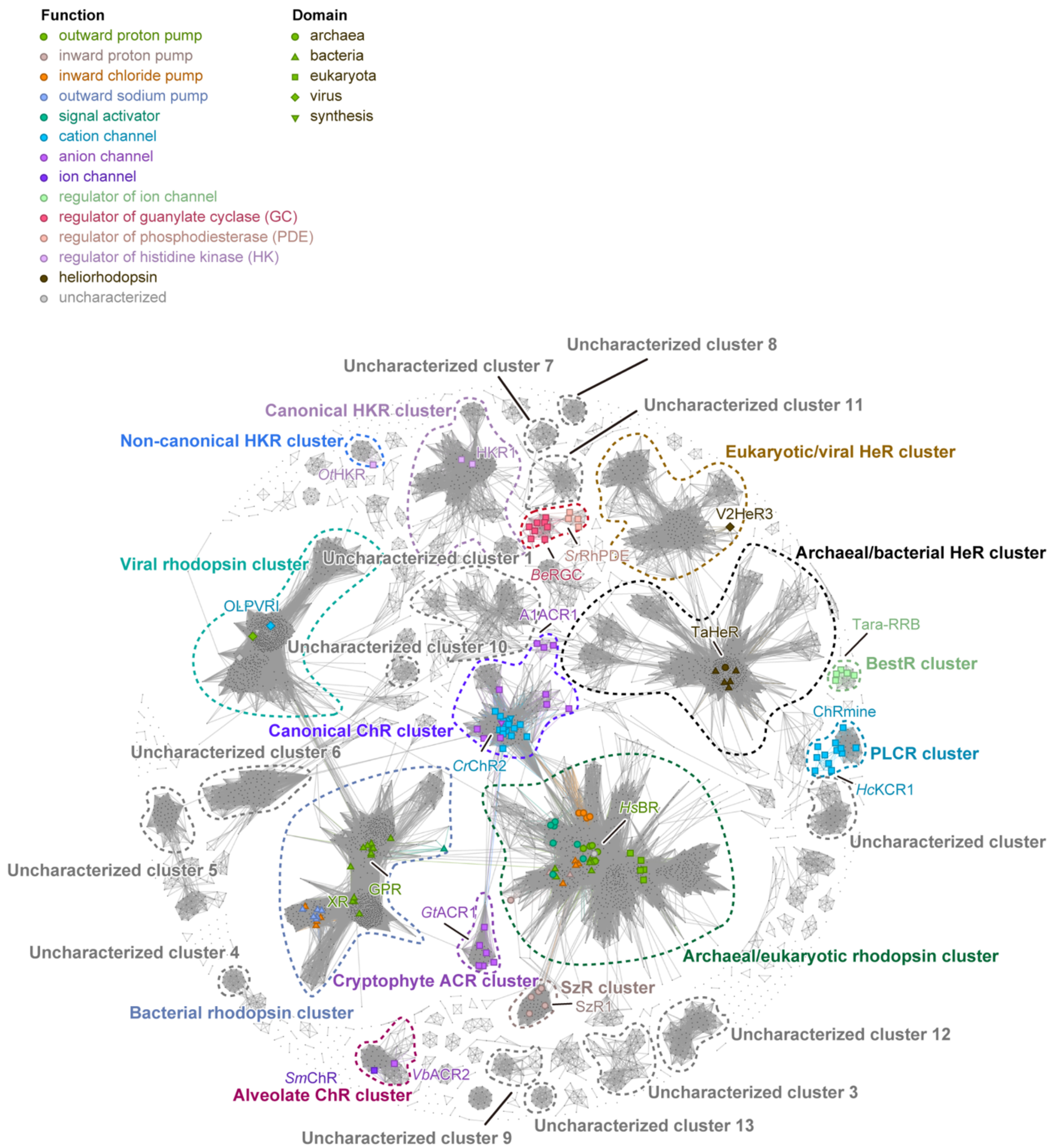

Supplementary Fig. 5 | Sequence similarity network of microbial rhodopsins. Sequence similarity network composed of approximately 40,000 non-redundant microbial rhodopsin sequences, including a large collection of uncharacterized rhodopsin-like sequences. Six major and eight minor clusters containing functionally characterized rhodopsins, as well as 13 clusters containing only uncharacterized rhodopsins, are highlighted with dashed circles. Representative rhodopsins are mapped onto the clusters, and the colors and shapes of the labels indicate function and domain (biological taxonomy), respectively.

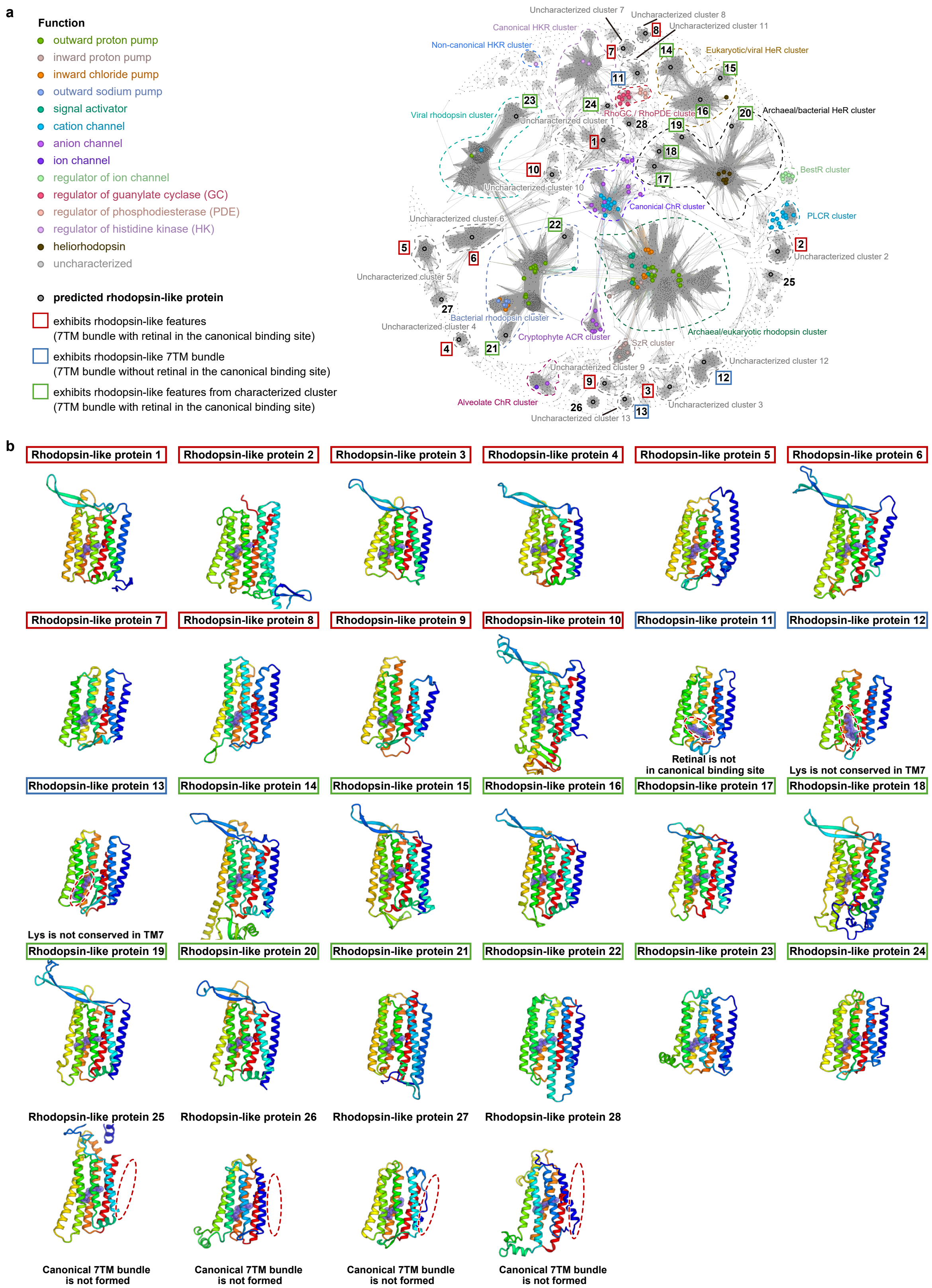

Supplementary Fig. 6 | Selection and predicted structures of rhodopsin-like sequences sampled from cluster peripheries. a Sequence similarity network composed of approximately 40,000 non-redundant microbial rhodopsin sequences, including a large collection of uncharacterized rhodopsin-like sequences. Six major and eight minor clusters containing functionally characterized rhodopsins, as well as 13 clusters containing only uncharacterized rhodopsins, are highlighted with dashed circles. Representative rhodopsins are mapped onto the clusters, and the colors and shapes of the labels indicate function and domain (biological

taxonomy), respectively. In addition, 28 rhodopsin-like sequences selected from the peripheral regions of characterized and uncharacterized clusters are highlighted. **b** Predicted structures of the 28 rhodopsin-like sequences selected from the peripheral regions of characterized and uncharacterized clusters. Four of the 28 sequences (25–28) exhibit a non-rhodopsin fold, one (11) shows non-canonical binding of the retinal chromophore, and two (12 and 13) lack the conserved lysine on TM7 that covalently binds the retinal chromophore.

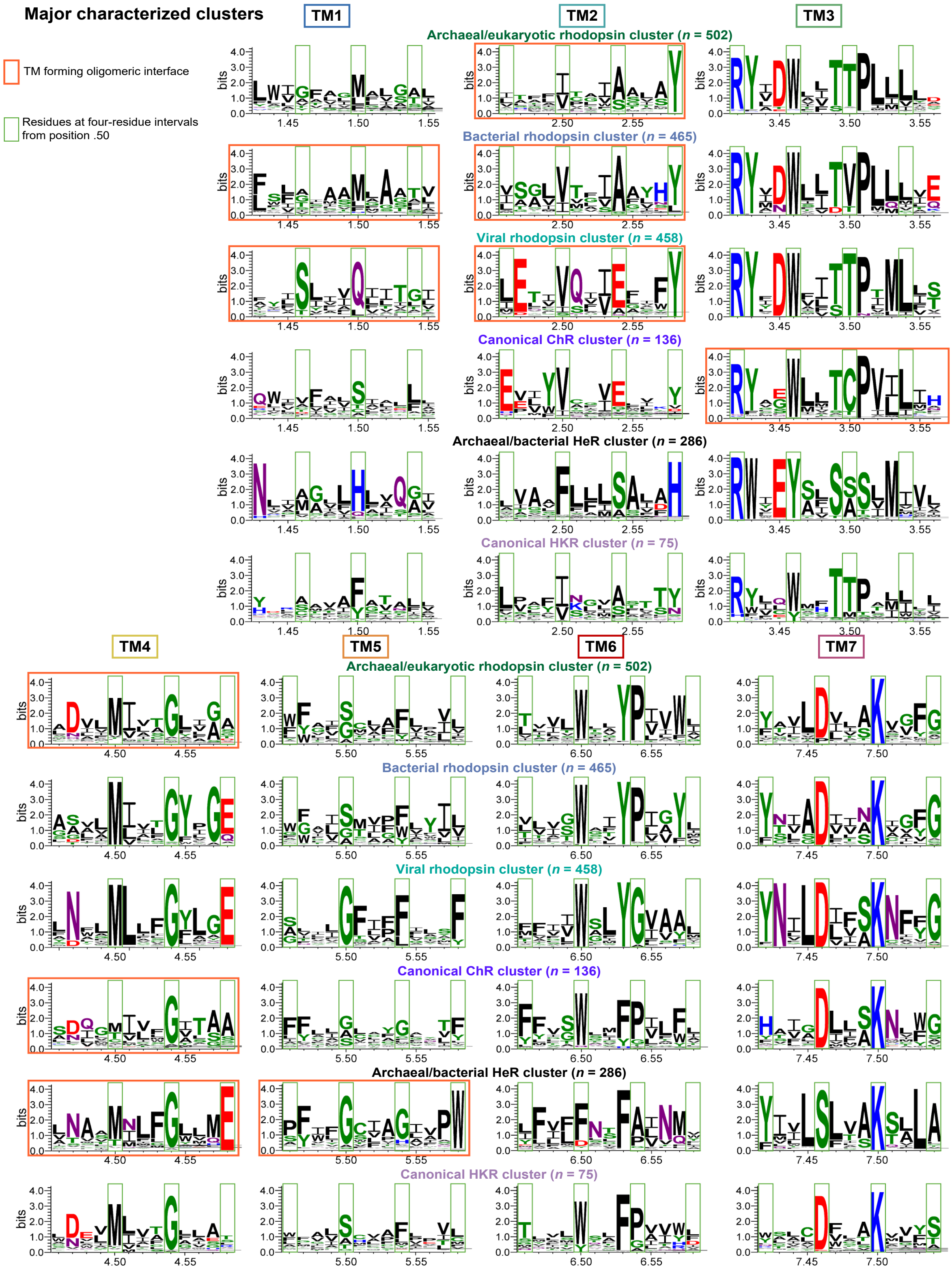

Supplementary Fig. 7 | Sequence logos of TM1–TM7 for the six major clusters containing functionally characterized rhodopsins. Transmembrane helices forming oligomeric interfaces and residues at four-residue intervals from position x.50 are highlighted with orange and green rectangles, respectively.

Minor characterized clusters

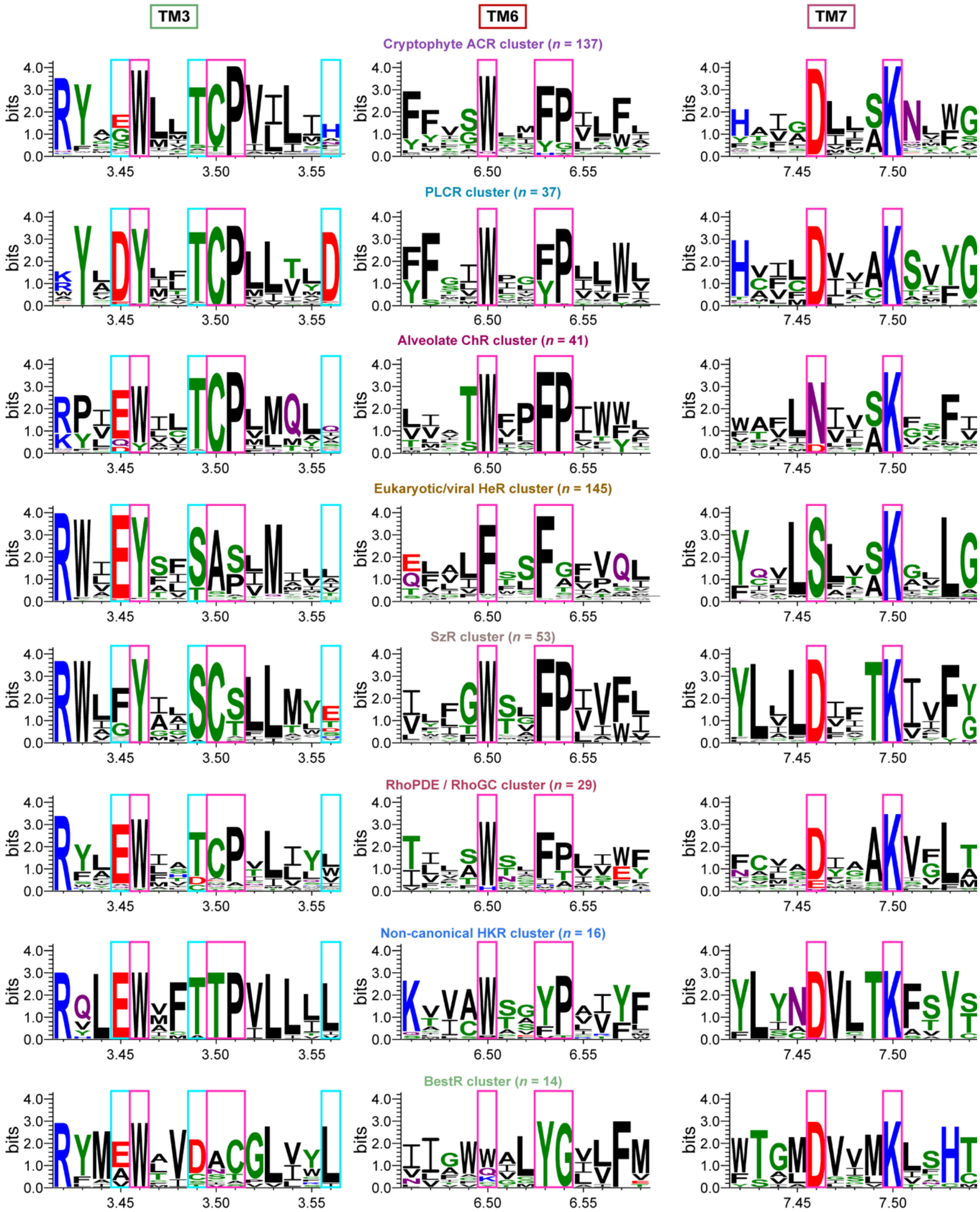

Supplementary Fig. 8 | Sequence logos of TM3, TM6, and TM7 for eight minor clusters containing functionally characterized rhodopsins. Cyan rectangles indicate residues of the DTD motif, and magenta rectangles indicate highly conserved residues within each cluster.

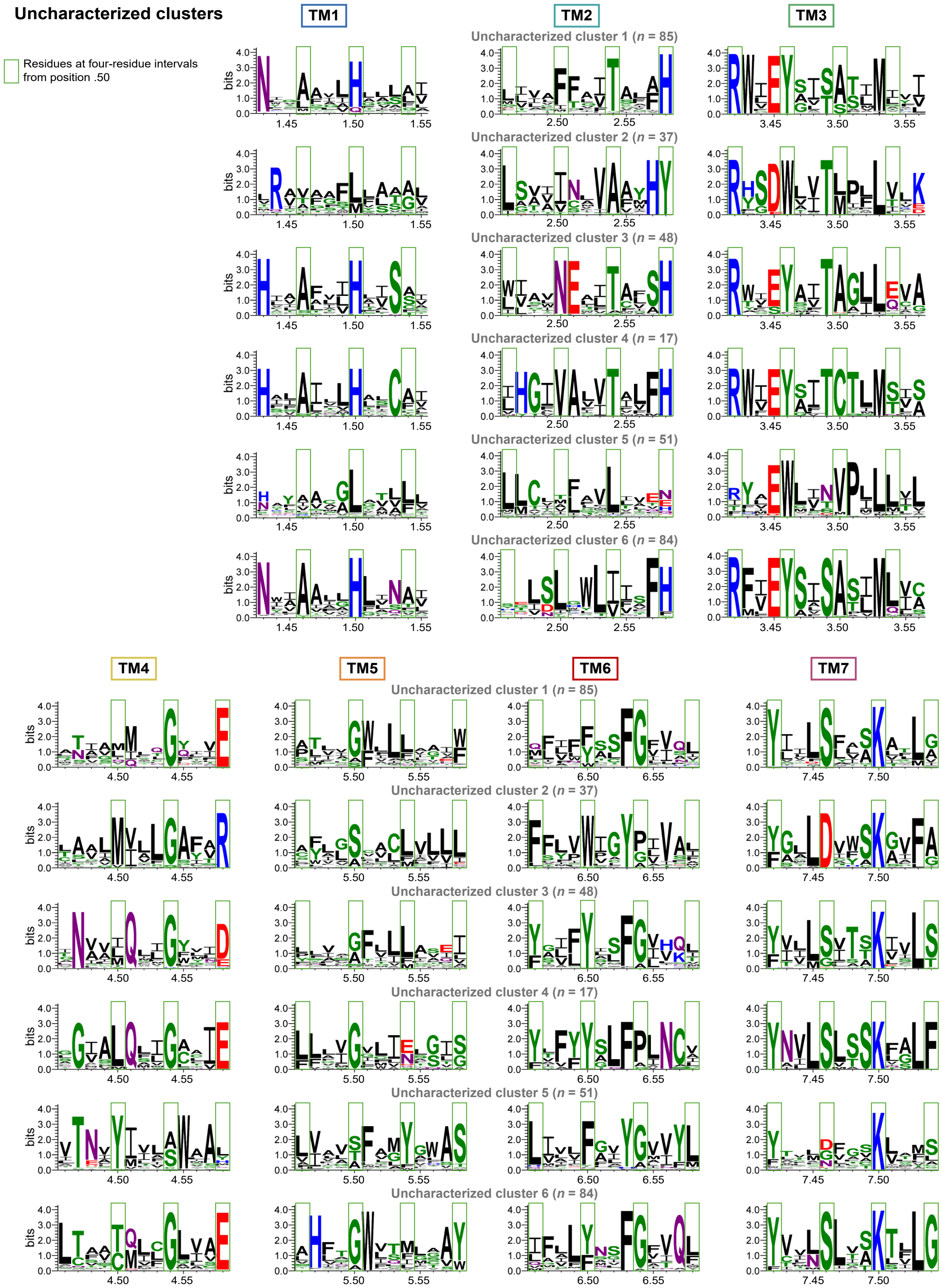

Supplementary Fig. 9 | Sequence logos of TM1–TM7 for the six uncharacterized rhodopsin clusters. Residues at four-residue intervals relative to position x.50 are highlighted with green rectangles.

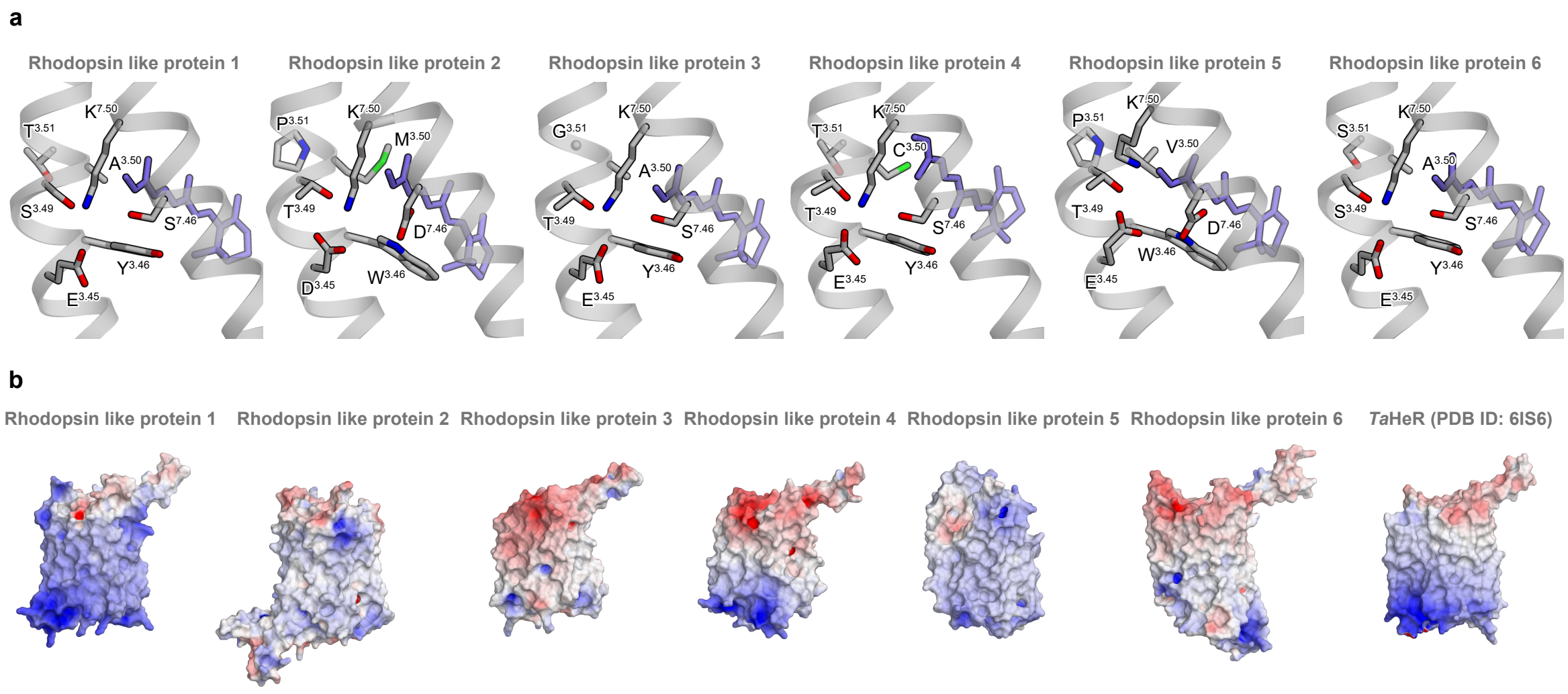

Supplementary Fig. 10 | Analysis of predicted structures of representative rhodopsins from six uncharacterized clusters. a Schiff base region of representative rhodopsin from the six uncharacterized clusters. **b** Surface electrostatic potential of representative rhodopsins from six uncharacterized clusters.

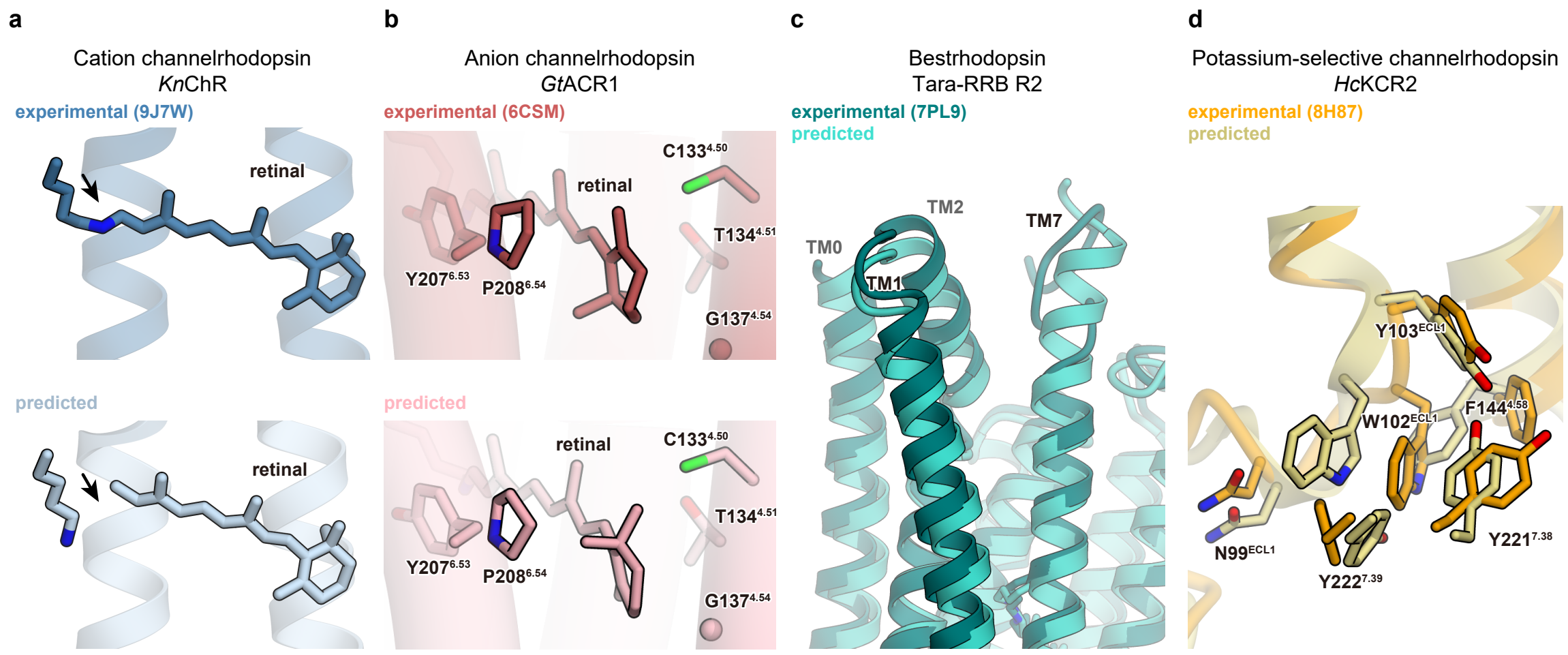

Supplementary Fig. 11 | Current limitations of structural predictions. Representative examples highlighting structural discrepancies between experimentally determined and computationally predicted structures. **a** In the predicted structure of *Kn*ChR, the retinal is not covalently linked to the Schiff base lysine. **b** In *Gt*ACR1, the β-ionone ring of the retinal adopts an incorrect twisted conformation. **c** In Tara-RRB R2, the cytoplasmic ends of TM0, 1, 2, and TM7 are poorly aligned. **d** In *Hc*KCR2, the architecture of the potassium selectivity filter is incorrectly predicted.
